# Free Radical Scavenging Activity and Inhibition of Enzyme-Catalyzed Oxidation by *trans*-aryl-Palladium Complexes

**DOI:** 10.3390/molecules30051122

**Published:** 2025-02-28

**Authors:** Koffi Sénam Etsè, Mohamed Anouar Harrad, Kodjo Djidjolé Etsè, Guillermo Zaragoza, Albert Demonceau, Ange Mouithys-Mickalad

**Affiliations:** 1 Laboratory of Macromolecular Chemistry and Organic Catalysis, Department of Chemistry, University of Liège, Sart-Tilman (B.6a), 4000 Liège, Belgium; ks.etse@yahoo.com (K.S.E.); a.demonceau@uliege.be (A.D.); 2 Laboratory of Medicinal Chemistry, Center for Interdisciplinary Research on Medicines (CIRM), University of Liège, Quartier Hôpital B36 Av. Hippocrate 15, 4000 Liège, Belgium; 3 Environmental, Ecological, and Agro-Industrial Engineering Laboratory, Sultan Moulay Slimane University, P.O. Box 523, Beni Mellal 23000, Morocco; ma.harrad@yahoo.fr; 4 Regional Centre for Education Training and Formation—CRMEF, Marrakech-Safi 40000, Morocco; 5 Laboratoire de Physiologie et Biotechnologie Végétales (LPBV), Faculté des Sciences (FDS), Université de Lomé (UL), Lomé BP 1515, Togo; kodjo.etse@yahoo.fr; 6 Unidade de Difracción de Raios X, Universidade de Santiago de Compostela, Edificio CACTUS, Campus Vida, 15782 Santiago de Compostela, Spain; g.zaragoza@usc.es; 7 Center for Oxygen, Research and Development (CORD), Center for Interdisciplinary Research on Medicines (CIRM), Veterinary Clinic, University of Liège, Quartier Vallée 2, Avenue de Cureghem 5, Sart-Tilman (B.6a), 4000 Liège, Belgium

**Keywords:** palladium, Hirshfeld surface, antiradical, efficiency, DPPH, ABTS, HRP, chemiluminescence

## Abstract

Herein, nine square planar *trans*-arylbis(triphenylphosphine)palladium halides (PdX(PPh_3_)_2_Ar) were synthesized and fully characterized. The molecular structure of two complexes (**1** and **2**) have been determined by both X-ray diffraction and described thanks to Hirshfeld surface analysis. Investigation of the antioxidant activities showed that most of the complexes exhibit a strong dose-dependent radical scavenging activity towards DPPH radical as well as in the ABTS radical scavenging test. Complexes **1** [PdI(PPh_3_)_2_(4-MeOC_6_H_4_)] and **3** [PdCl(PPh_3_)_2_(4-MeOC_6_H_4_)] showed the highest activity in the DPPH assay with EC_50_ values of 1.14 ± 0.90 and 1.9 ± 0.87 µM, respectively. In contrast, for the ABTS assay, quercetin (5.56 ± 0.97 µM) was slightly more efficient than the three complexes **1** (5.78 ± 0.98 µM), **2** (7.01 ± 0.98 µM), and **3** (11.12 ± 0.94 µM). The use of kinetic studies as a powerful parameter shows that complexes **1**, **2**, and **3** displayed the best antioxidant efficiency. The antioxidant effect of the nine palladium complexes has been also evaluated on the enzyme-catalyzed oxidation of the L012 probe (using HRP/H_2_O_2_) by using a chemiluminescence technique. As with the last model, complexes **1**, **2**, and **3** showed the best activity, with EC50 values of 3.56 ± 1.87, 148 0.71, and 5.8 ± 2.60 µM, respectively. Interestingly, those complexes (**1**, **2**, and **3**) even exhibited a higher dose-dependent activity than the quercetin (7.06 ± 2.56 µM) used as a standard. Taken together, the combined results reveal that the antiradical and enzyme (HRP) inhibitory activity of complexes decrease following the ligand order of *p*-OMePh > *p*-OAcPh >> Ph.

## 1. Introduction

Inflammation is one of the main causes of several diseases and is considered a key factor triggering the development of diseases, such as atherosclerosis, diabetes, and cancer [1,2,3,4,5,6,7,8]. One of the potential targets for new drugs could be the redox process widely involved in the development of cancer. Indeed, during inflammation, a redox cycle takes place and triggers oxidative processes by enhancing the production of reactive oxygen species (ROS)/free radicals. On the other hand, it is well-known that immune cells, like polymorphonuclear neutrophils, play a crucial role in oxidative stress. The worldwide cancer incidence is still increasing, with particularly high levels of death in Western countries, causing a real health problem with social costs [9,10]. Among the current treatments for cancers, one can find surgery, radiotherapy, chemotherapy, hormonotherapy, immunotherapy, and new “targeted” treatments [11,12,13,14]. Furthermore, special attention has been focused on the biological activity of the various metal ions or organometallic compounds used in metal-based drug therapy. The most widely used compound in this therapy is cisplatin [15,16,17,18]. Although its beneficial effects on certain types of cancer are well-known, it is nonetheless true that this compound has a known toxicity on kidneys and that patients often develop resistance to it, especially in the case of solid cancers [14,19]. 

The current challenge for chemists is to find new molecules with low toxicity and a good affinity with carriers or biomolecules, such as albumin, transferrin, and others that display a high affinity for cancer cells [20]. Data from the literature indicate that some palladium (Pd) and platinum (Pt) complexes could have this property and, therefore, display interesting therapeutic effects [21,22,23,24]. For that purpose, a variety of Pd-based complexes were designed and synthesized for new targeted therapeutics [25,26,27]. In addition, palladium-based complexes, bearing various ligands, have been proposed and tested on various cancer cell lines and others for their anti-fungal, anti-microbial, or yeast antiviral effects [28,29,30]. Indeed, a direct link between cancer and inflammation and redox process through ROS has been proven [31] with the inhibition of the NADPH oxidase enzyme [32,33]. Nevertheless, during inflammatory and stress conditions, like sepsis, excessive activation of NADPH oxidase induces the production of high concentrations of ROS, amplifying the oxidation, nitration, and chlorination of some molecules of interest, such as DNA, proteins, etc. [34,35,36], which could lead to the occurrence of cancers. 

Due to the link between inflammation, oxidation, and various pathologies, it is important for us to perform a preliminary study on the antioxidant activity of these bio-organometallic complexes. The anti-ROS activity coupled with the cytotoxic proprieties of these molecules should be an extremely effective synergy in the treatment of several diseases.

To implement a model of screening for candidate molecules, a biochemical approach was performed using horseradish peroxidase (HRP), which works in similar manner to human myeloperoxidase (MPO). In the presence of H_2_O_2_, HRP forms the radical cation [P-Fe ^IV = 0^]•^+^ that will attack the luminol analog, L-012 (8-amino-5-chloro-7-phenylpyrido [2,4-d]pyridazine-1,4(2H,3H)-dione), to emit detectable and measurable luminescence [37,38]. Our interest in using L-012 comes from the fact that it produces a much stronger chemiluminescence than lucigenin, luminol, and MCLA (2-Methyl-6-(4-methoxyphenyl)-3,7-dihydroimidazo [1,2-a]pyrazin-3-one) and because it is not subject to redox cycling [39,40]. This latter property makes L-012 an ideal probe for reactions involving metals [41].

The palladium complexes of the generic formula *trans*-[PdX(PPh_3_)_2_(4-RC_6_H_4_)] where X is halogen and R any substituent, are well-known and widely used for their catalytic activity [42]. In addition, our group recently reported the redox cycling ability of these complexes and showed their potency as new materials for a Li-ion battery [43,44]. This family of complexes is prepared by the oxidative addition of organic halides of sp^2^ carbons on Pd(PPh_3_)_4_, and the rate of the addition decreases in the following order: C-I > C-Br >>> C-Cl >>> C-F. Thus, other halide (F, Cl, Br) complexes are generally synthesized from the iodinated complexes using different halogen sources [45]. 

The present work aimed to first investigate the structural description of the *trans*-[PdX(PPh_3_)_2_(4-RC_6_H_4_)] complexes and then their radical scavenging activity. Therefore, ABTS and DPPH methods were used as chemical models, and a kinetic study via a DPPH assay was performed to better understand the reactivity and efficiency of the complexes studied. Finally, the inhibitory effect of different complexes was assayed on the enzyme-catalyzed oxidation of an L012 probe monitored by a chemiluminescence (CL) assay.

## 2. Results and Discussion

### 2.1. Synthesis and Characterization of Palladium Complexes

**Scheme 1:**
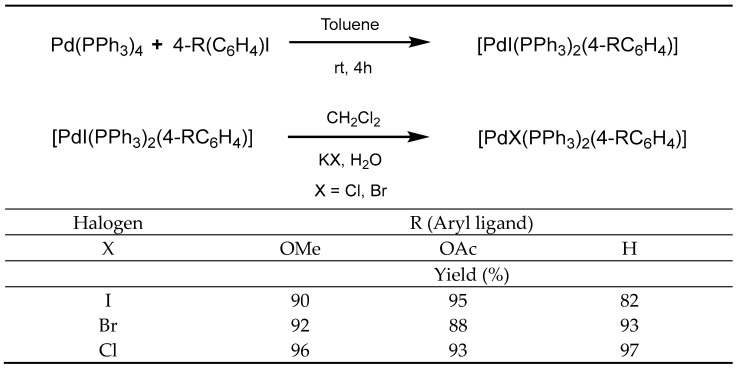
Preparation procedure and yields of complexes [PdX(PPh_3_)_2_(4-R-C_6_H_4_)].

The preparation of the *trans*-arylbis(triphenylphosphine)palladium halides [PdX(PPh_3_)_2_(4-R-C_6_H_4_)] (with R = H, OMe, OAc and X = Br, Cl) was performed based on the initial method reported by Flemming et al. [45] and Etse et al. [44]. First, the iodine derivatives were obtained by oxidative addition of 4-R-C_6_H_4_I onto Pd(PPh_3_)_4_ (Scheme 1). The reactions were performed in toluene with total conversion in 4h. The products precipitated from reaction mixtures. The different complexes [PdI(PPh_3_)_2_(4-R-C_6_H_4_)] were isolated with high yields (Scheme 1) after filtration on a Büchner funnel, drying under vacuum, and storage under a nitrogen atmosphere. 

The chloride and bromide derivative complexes were prepared from their iodide analogs. The [PdI(PPh_3_)_2_(4-RC_6_H_4_)] complexes were dissolved in dichloromethane and washed repeatedly with saturated solutions of potassium halide (KBr or KCl) in order to induce halogen exchange. The reactions were monitored by ^31^P NMR until a complete conversion was reached.

Thanks to this procedure, the six complexes with the generic formula [PdX(PPh_3_)_2_(4-RC_6_H_4_)] (with R = H, OMe, OAc, and X = Cl or Br) were obtained and fully characterized.

After the synthesis, the nine complexes were characterized using ^1^H, ^13^C, and ^31^P NMR techniques. The ^1^H, ^13^C, and ^31^P NMR spectra of complexes **4** to **9** agree with the data previously reported, confirming their structure [44,45]. Special attention is, therefore, focused on the characterization of the new complexes **1**, **2**, and **3**. The ^31^P NMR spectra showed only one peak confirming the exclusive formation of the *trans* complexes. These peaks are observed on the ^31^P NMR spectra at around 22.77, 23.41, and 23.54 ppm for complexes **1**, **2**, and **3,** respectively. The proton NMR spectra show a singlet at 3.48, 3.53, and 3.49 ppm, revealing the presence of methyl group of the methoxy substituent in the structure of compounds **1**, **2**, and **3,** respectively. Finally, in addition to the multiplet corresponding to an integration of 30 protons that can be observed between 7.2 to 7.7 ppm, characteristic to triphenylphosphine ligands, 2 doublets are also observed around 6.0 and 6.6 ppm, attesting to the presence of the hydrogen atoms in the *ortho* and *meta* positions of the aromatic ring linked to palladium metal center. All the NMR spectra are available in the Supplementary Materials.

### 2.2. Mass Spectrometry Analysis

Upon electro-spray ionization, the synthesized neutral palladium complexes **1**–**9** lost their halide ligand, resulting in [Pd(PPh_3_)_2_(4-RC_6_H_4_)]^+^ pseudo-molecular ions, indicating that ionization of these compounds took place via the loss of the halogen ligand. These results agreed with our previous observations during the analysis of complexes **4**–**6** [43]. Furthermore, these species are decomposed into metal-free [P(Ph)_2_(4-RC_6_H_4_)]^+^ cations. These results are in accordance with earlier stability studies on this family of complexes conducted by different authors [46,47,48,49] showing the formation of a new phosphine derivative because of aryl–aryl exchange during decomposition and rearrangement. Complexes bearing chloride ligands, namely **3**, **6**, and **9**, were more exposed to this exchange.

### 2.3. X-Ray Diffraction Analysis

To complete the structural characterization of the prepared complexes, X-ray diffraction analysis has been realized. Since our group has already reported the molecular structures of complexes **4**, **5**, and **6 [44]** and Flemming’s group has reported the structures of complexes **7**, **8**, and **9** [45], our current attempts are, therefore, focused on only complexes **1**, **2**, and **3**. 

Various attempts to obtain a crystal suitable for X-ray diffraction analysis were carried out for compounds **1**, **2**, and especially for **3**. For compound **3**, in most cases, the solution became brown or black without the formation of a crystal, indicating the decomposition and degradation of the complex. Fortunately, yellow crystalline needles suitable for X-ray diffraction analysis were obtained by slowly cooling solutions of complexes **1** and **2** in dichloromethane. The crystal data, data collection, and structure refinement details of complexes **1** and **2** are summarized in Table 1.

**Figure 1:**
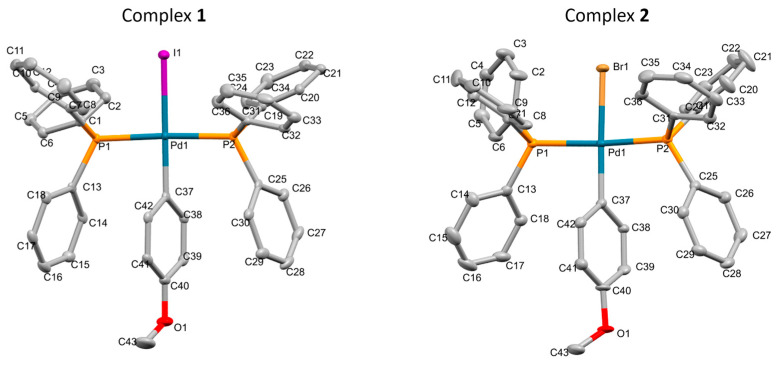
ORTEP representation of complexes **1** and **2** with thermal ellipsoids drawn at the 50% probability level (hydrogen atoms are omitted for the sake of clarity).

The molecular structures of both complexes (**1** and **2**) were, therefore, confirmed by X-ray diffraction analyses (Figure 1). Complex **1** crystallizes in the monoclinic, *Ia* space group, whereas complex **2** crystallizes in orthorhombic, *Pbca* space group. Selected bond distances and angles are reported in Table 2 to compare the geometrical parameters of the two structures. The two triphenylphosphine ligands are positioned in the *trans* position. The two palladium complexes present a square planar geometry (Figure 1), a well-known molecular structure for this class of complexes [44,45]. As expected, the palladium–halide bond length is short in complex **2** compared to complex **1** with 2.5120 (5) **Å** and 2.7009 (3) **Å,** respectively. Other selected bond lengths are in the same range. Concerning the specific bond’s angle, the values of C(Ar) –Pd1–P observed for complex **1** (~88.60°) are lower than those obtained for complex **2** (~91.63°). In contrast, the value of the P–Pd–X angle is larger in complex **1** compared to complex **2**. Consequently, the value of the P2–Pd1–P1 angle in **1** (176.86 (3)°) is greater than the one obtained in **2** (173.39 (4)°), because the *trans* influences the tendency of halogens generally observed due to the iodine atom size.

### 2.4. Hirshfeld Surface Study of Complexes ***1*** and ***2***

**Figure 2:**
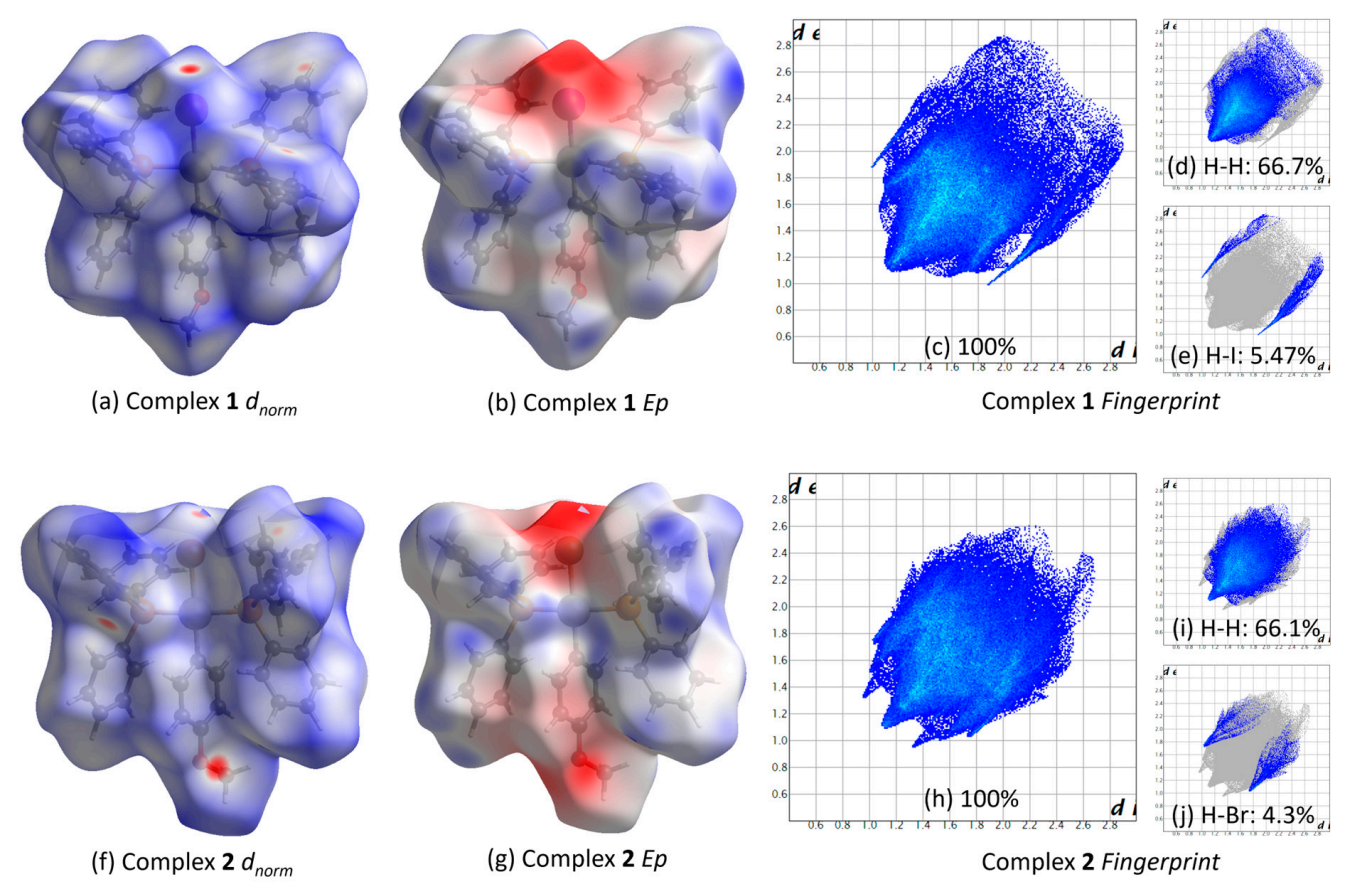
The Hirshfeld surface of complexes **1** and **2** mapped over *d_norm_* (**a**,**f**) and molecular electrostatic potential (**b**,**g**). Two-dimensional global (**c**,**h**), H⋅⋅⋅H (**d**,**i**), and H⋅⋅⋅halide/halide⋅⋅⋅H (**e**,**j**) decomposed atom pairwise fingerprint plots of complexes **1** and **2**.

In view of completing the description of the crystal structure of the complexes presented above, calculation, description and analysis of their Hirshfeld surface (HS) were realized. The CrystalExplorer 17 program was used to generate these surfaces and to analyze them according to the literature procedure [50]. The HS of the two compounds mapped over descriptors *d_norm_* and molecular electrostatic potential are presented in Figure 2. The surfaces are transparent to enable easier visualization and identification of the atoms involved in the different interactions. In this study, the *d_norm_* value ranges from −0.1254 to +1.4075. 

For complex **1**, a red spot is observed on the top around the iodine atom. The red spots on the surfaces mapped over *d_norm_* reveal short contacts (Figure 2a). This observation clearly suggests that the main interatomic contact is established with the iodine atom, in accordance with the interaction analysis between the iodine and one hydrogen (*H*43B) of the *OCH_3_* atoms performed and reported in Figure S28. That contact is characterized by the I–H distance of 2.99 and I–H*^i^*43B–C*^i^*43 angle of 169.2°. The similar position of a red spot around the bromine atom is not clearly observed during the analysis of compound **2**, since it appears that the Br is positioned at the summit of a tetrahedron formed for the hydrogen atom of three different neighbors’ structures. In contrast, a red spot is located around the oxygen atom of the methoxy group, suggesting the presence of a hydrogen bond (Figure 2f). Deep analysis showed the existence of a hydrogen bond with another molecule of the crystal packing the following parameters: O1–H9*^ii^* = 2.424 and C9*^ii^*–H9*^ii^*–O1 = 170.41° (Figure S29).

The electrostatic potential for compounds **1** and **2** are calculated at the B3LYP/6–31G(d,p) level of theory and mapped on the Hirshfeld surface in the range from −0.0710 to + 0.0338 a.u (Figure 2b,g). For the electrostatic potential surface on HS, the electron-rich and electron-deficient sites in the molecule can be observed as blue and red regions corresponding to positive and negative electrostatic potentials, respectively. The results show that, for complex **1**, the electronegative region is mainly centered on the iodine where the surface is concave, highlighting the electronegative property and the size effect of the iodine atom (Figure 2b). For complex **2**, the electronegative regions are observed in two regions. The first region is located around the bromine, and the second region is located around the oxygen atoms (Figure 2g). These results suggested that the larger size of the iodine atom strengthens the triphenylphosphine ligands that go downward, leading to steric hindrance around the aryl ligand in contrast to the bromine atom. Consequently, the 4-OMePh- ligand in complex **2** is more exposed, allowing the establishment of a hydrogen bond.

The geometry adopted by the two compounds leads to specific contacts in the crystal packing that could be effectively described by analyzing their molecular fingerprint. Indeed, the distances from the HS to the nearest nucleus inside the surface (*d_i_*) and outside the surface (*d_e_*) are used to create a 2D histogram, called a fingerprint plot [51]. The fingerprint of a considered molecule is unique since it is highly sensitive to the immediate environment and contact of the molecule [52]. The molecular FP of the two complexes is presented in Figure 2c,h, showing notable discrepancy between the two molecules. Since the only difference between the two molecules is the halogen atom, the discrepancy observed can be explained by the halide effect and crystal packing. As the fingerprint plot can be used to evaluate the contributions of interatomic contacts to the HS, the decomposition of the fingerprint pair wise component is used to identify and quantify the weight of specific contacts. The results reveal that for compounds **1** and **2**, the H···H contacts contribute 66.7% and 66.1%, respectively, and appear as the dominant intermolecular interactions. Interestingly, the only contact observed with the halogen in the packing is with the hydrogen atoms. For complexes **1** and **2,** the results for H···I/I···H and H···Br/Br···H were 5.47% and 4.3%, respectively. This result is in alignment with the iodine atom size’s effect, enhancing the contact percentage. The difference between these two types of contacts is clearly visible when mapping the decomposed FP, as shown in Figure 2e,j. Finally, the presence of a red spot around the oxygen atom in complex **2** is confirmed by the H···O/O···H contact contribution value of 3.4%, which is higher than that observed for complex **1** (2.4%).

### 2.5. Effect of Palladium (II) Complexes on the Free Radical Scavenging Activity

**Figure 3:**
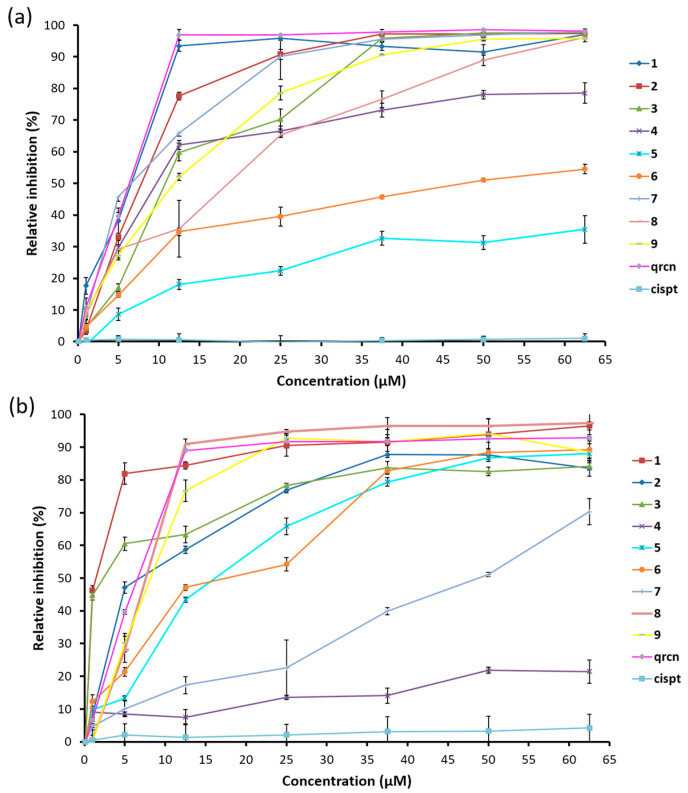
Kinetic inhibition curves of ABTS^•+^ (**a**) and DPPH^•^ (**b**) obtained in the presence of increasing concentrations of Pd-based complexes compared to quercetin and Cis-Pt (taken as antioxidant or organometallic reference molecules, respectively).

The preparation and antioxidant activity of various metal complexes have been reported [53,54,55,56,57,58,59]. In the frame of this report, the palladium (II)-based complexes that were prepared and characterized were first studied for their potential free radical scavenging activity using two well-known chemical assays (ABTS and DPPH). Using these two techniques, we found that complex **1**, bearing a *para* methoxyphenyl moiety, showed the best antiradical activity with an EC_50_ = 5.78 µM, compared to the reference molecule quercetin (**Qrcn**) with an EC_50_ = 5.56 µM. It appears that the result obtained with **Qrcn** is lower than the values reported in the literature, which are around 15 µM [60]. All the results are presented as values of EC_50_ (Table 3). The least effective antioxidant compound against ABTS^•+^ is complex **5**, with an EC_50_ = 0.135 mM. Three different groups can be considered depending on the nature of substituent R: the first group is composed of molecules where R can be methoxy, while R can acetoxy for the second group, and R can be a hydrogen atom for the last group. Complexes of the first group are more active against the ABTS radical cations. This difference is probably due to the presence of the methoxy group which enhances their antiradical activity, as described by different authors [60,61]. Regarding the role of the halide atom attached to the palladium in these compounds, it appears that the scavenging activity follows the following order: I > Br > Cl. This result can be explained by the chemical property of halide, which is a leaving group, thus causing an increase in the scavenging activity. Cisplatin (CisPt), which has two chlorine atoms in its structure, does not show any radical scavenging activity against the ABTS^•+^ (Figure 3a).

Knowing that individual antioxidant molecules are more efficient at quenching certain radicals than others, it was interesting to confirm the results obtained with the ABTS test and the DPPH method. The DPPH radical test is based on an electron transfer (ET) mechanism and should give new information about reaction pathways. The two ABTS and DPPH results are further compared to establishing their consistency across different complexes. In contrast to the ABTS assay, the EC_50_ values found in the DPPH test for all the compounds, except for complex **4**, were much lower than that seen for resveratrol (74 µM [62]). As expected in the DPPH^•^ radical scavenging test, complex **1** exhibited the best scavenging activity (EC_50_ = 1.14 µM). Surprisingly, and contrary to the ABTS test, complex **3** showed very good activity with an EC_50_ = 1.9 µM, slightly higher than that of compound **1** (EC_50_ = 1.14 µM). Compared with flavonoids, such as epicatechin with an IC_50_ = 15.7 µM, our complexes appear to be more efficient [63]. It was demonstrated that ABTS^•+^ is also soluble in aqueous and organic solvents and is not affected by ionic strength; therefore, it is used for both its hydrophilic and lipophilic antioxidant capacities [64]. These considerations allow us to state that the mechanisms of action of the complexes against both ABTS^•+^ and DPPH^•^ radicals are not identical. Compounds bearing the *para*-methoxyphenyl group are more hydrophobic than those bearing the *para*-acethoxyphenyl one, regardless of their poor solubility in methanol, DMSO, and water.

In the DPPH test, compound **4** (IC_50_ = 1.84 mM) showed a very low radical scavenging reactivity compared to the ABTS assay, where it showed relatively good activity (EC_50_ = 10.89 µM, Table 3). This discrepancy can be explained by the pathway by which the compound reacts with both radicals (ABTS and DPPH). The interactions between the antioxidants and DPPH^•^ are also determined by the antioxidant’s structural conformation. It was shown that the conformation of compound **3** rapidly changes in the presence of a solvent, resulting in *para*-methoxyphenyl group migration to the phosphorus atom. This observation was never reported in the case of compound **4** at room temperature and in such a short period but has been reported in the case of complex **9** at relatively high temperature. Nevertheless, the antioxidant activity of complexes **8** and **9** is better than that seen for compounds **4**, **5**, and **6**. In the ABTS assay, like in the DPPH tests, the CisPt inhibition curve did not converge; therefore, the EC_50_ values could not be calculated. CisPt shows no antioxidant activity.

### 2.6. Antioxidant Efficiencies

To evaluate antioxidant efficiency, it is important to use a parameter that allows us to make the comparison. Therefore, expressing the EC_50_ value as the weight of molecules in grams necessary to inhibit a kilogram of DPPH radicals seems to be a good estimation of a molecule’s efficiency. The EC_50_ values of compounds **1** and **3** were 50.27 and 75.06 g of antioxidant per Kg of DPPH^•^, respectively. The two values are inferior to those obtained for quercetin (87.49g/Kg) compared to the literature (84 ± 6 g/Kg) [54]. In contrast, the use of other ligands, such as acethoxyphenyl (**4**, **5**, and **6**) and phenyl (**7**, **8**, and **9**), instead of a methoxyphenyl one, resulted in a decrease in the activity (Table 3). The interaction mechanism of metal complexes with free radical sources can proceed toward various processes. It was apparent that antioxidants could attack free radicals by using one of three mechanisms or their combination, namely hydrogen atom transfer [HAT], single-electron transfer followed by proton transfer [SET-PT], and sequential proton loss electron transfer [SPLET]. Making a clear distinction between these processes is difficult, because various mechanisms may occur during these processes, leading to a cascade of mechanisms. In addition, it was reported that there was an additional process where spin entrapment could happen. Finally, the mechanism depends on the antioxidant’s structure, solubility, stability, and experimental conditions, including pH, solvent, and temperature [65]. For the complexes studied, the results of the mass spectrometry analysis show that the complexes decompose by losing the halogen to form a palladium pseudo-molecular ion, which then decomposes into metal-free cations. In addition, aryl–aryl exchange followed by rearrangement by-products was observed in solution for these complexes (see Figure S30: unpublished work). These results suggest a dynamic decomposition/rearrangement possibility of the complexes in solution. According to Grushin, the mechanism of the aryl–aryl exchange reactions in noncoordinating solvents of low polarity of these compounds may not require Pd-X ionization but might instead involve phosphine dissociation involving a tight ion pair intermediate [66]. In this study, most of the complexes interact very quickly with DPPH. Considering the rate of the reaction, we can reasonably propose that the first step of the reaction is the reduction of DPPH due to the electron transfer (ET) mechanism from the palladium pseudo-molecular ions [67]. This hypothesis agrees with the fact that the DPPH assay is mainly based on an ET mechanism, as the hydrogen-atom transfer (HAT) or abstraction can be considered as a marginal reaction pathway in this case [68]. It is admitted that the accessibility of the DPPH radical center and side reactions induced by molecules play an important role and then lead to the HAT mechanism [68]. On the other hand, it is possible to establish the correlation between the reactivity of the tested compounds and their molecular volume values. Via X-ray diffraction analysis, it was possible to obtain those values for complexes **1** and **2**, which were 3578.6 Å^3^ and 7116.3 Å^3^, respectively, showing that the volume of complex **1** was two times lower versus complex **2**. This parameter can enhance the accessibility and the interaction of complex **1** towards the DPPH radical center. Although the EC_50_ values of compounds **1** and **3** are closer, that of compound **2** is six times higher. 

To better understand this result, we decided to use the efficiency factor of an antioxidant as defined by Sanchez-Moreno [69]. This parameter basically gives a more precise idea of the antiradical efficiency (AE), which involves the potency (1/EC_50_) and the reaction time (T_EC50_). The lower the EC_50_, the lower the T_EC50_ and the higher the AE. The results are presented in Table 4.

Although these six compounds showed a good radical scavenging activity (EC_50_ < 10 µM), their antiradical efficiencies are not exceptional. Apart from complex **1**, for which the activity can be classified as medium, all the other compounds are found to have low efficiency. Even though the EC_50_ value of compound **3** is very low (1.9 µM), at least 63 min is necessary for it to reach a steady state; as such, it has the lowest antiradical efficiency. Furthermore, although complex **8** expresses a good activity against DPPH radicals, with an EC_50_ = 6.64 µM, its efficiency is much lower than that of complex **2** (EC_50_ = 7.06 µM). After considering the molecule structures, the fundamental difference in the group of *para*-methoxyphenyl ligand complex holders remains the halogen bonded to the palladium. This last factor could justify the results obtained and might be responsible for their antiradical efficiency. Thus, the weak Pd–I-bound energy should increase its facile ionization and, thus, its antiradical power, since the reactivity of an antioxidant lies in the competition of these various reactions [70]. In the second family of compounds, with a *para*-acetoxyphenyl ligand, the radical scavenging activity is less important, and the T_EC50_ is too high, resulting in a very low efficiency.

### 2.7. Enzyme-Catalyzed Oxidation Monitored by Chemiluminescence (CL) Assay

**Figure 4:**
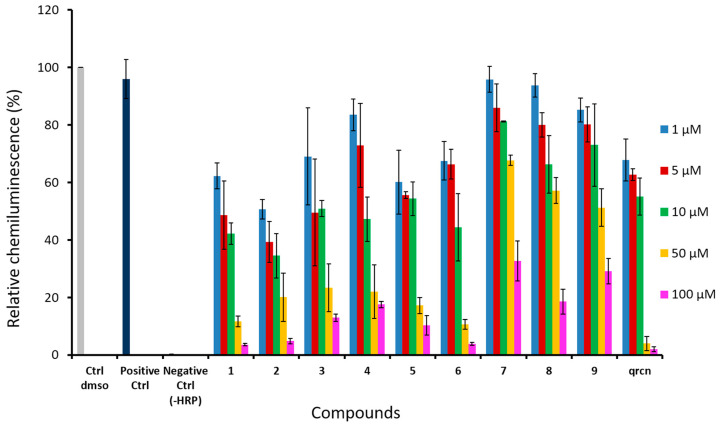
Inhibitory effect of increasing concentrations (1–100 µM) of Pd-based complexes (**1**–**9**) compared to **Qrcn** on the chemiluminescence produced by the HRP-H_2_O_2_/L-012 system.

The chemiluminescence test is based on the oxidation of the probes, which leads to radicals derived from the molecules producing excited-state species that emit light (chemiluminescence). Any compound able to react with radical initiators might inhibit or enhance the production of light. We have used this technique to study the antioxidant activity of the studied complexes to better understand their mechanism of action towards the enzymatic system, which is involved in the redox phenomenon and inflammation. The enzyme-catalyzed oxidation model using horseradish peroxidase (HRP) was employed based on the literature data [71]. Herein, the system HRP-H_2_O_2_ has been used as source of radical cations and L-012 has been used as a chemiluminescent probe. HRP reacts with hydrogen peroxide to give peroxidase species, such as the radical cation intermediate [P-Fe ^IV = 0^]^•+^, also named compound I. This latter compound reacts with L-012 to give rise to an excited intermediate and resulting light emission. Thus, in the presence of an antioxidant molecule, the latter one is oxidized by compound I, thereby inhibiting luminescence. Compared to chemical assays (ABTS and DPPH), our results obtained with the enzyme model (HRP-H_2_O_2_), using the CL technique, show that most of the complexes exhibited a variable dose-dependent effect on light emission (Figure 4). 

Among them, complexes **1** and **2** displayed a pronounced antioxidant activity with EC_50_ values of 3.56 and 1.48 µM, respectively. These results confirm those already observed (ABTS and DPPH tests). 

At the lowest concentrations of 1, 5, and 10 µM, complexes **4**, **7**, **8**, and **9** had a weak effect, likewise for quercetin. It is important to note that, at higher concentration of 100 µM, compound **7** displayed good activity against the ABTS radical and compound **8** displayed good activity against DPPH one. The two complexes showed less interesting antiradical scavenging properties in the enzymatic model with EC_50_ = 66.99 and 33.34 µM, respectively.

Complexes **1**–**6** with *para*-methoxyphenyl and *para*-acetoxyphenyl ligands showed a more interesting activity in the enzymatic system but those with phenyl ligand were less effective (Table 5). The activity of complexes can be classified according to the nature of their ligands, as follows: *para*-OMePh > *para*-OAcPh >> Ph. For the Ph and *para*-OAcPh ligands, the iodine complex is less of an inhibitor, followed by the chlorinated one and finally by brominated complexes. This result agrees with the order of the electro-attracting effects and electronegativity among the halogens. By contrast, for the other complexes, a general tendency of the halogen effect is not observed. It is, therefore, clear that the substituent in the *para* position on the aryl ring also plays an important role in the activity of the compounds. The main effect of the methoxy group would enhance the electron density of the aromatic cycle and, therefore, influence the metal, improving the complex’s activity. 

## 3. Materials and Methods

### 3.1. Materials and Physical Techniques

Analytical-grade methanol and ethyl acetate (Chem-Lab, Zedelgem, Belgium) were used in the extraction procedure. Sodium chloride (NaCl), potassium chloride (KCl), potassium bromide (KBr), ammonium acetate, potassium hydroxide, sodium hydroxide, acetic acid, ethanol, hydrogen peroxide (H_2_O_2_), and dimethyl sulfoxide (DMSO), were all purchased from Merck (VWRI, Leuven, Belgium). Pd(PPh_3_)_4_, *para*-iodomethoxyphenyl, *para*-acetoxyphenyl, iodobenzene, and toluene were obtained from Aldrich (Leuven, Belgium). ABTS (2,2- azinobis (3-ethyl- enzothiazoline-6-sulfonic acid) and DPPH (1,1-diphenyl-2-picrylhydrazyl) were purchased from Sigma-Aldrich (Steinheim, Germany). Quercetin (3,3′,4′,5,7-pentahydroxy-2-phenylchromen-4-one) was from ChromaDex (LGC Standard, Molsheim, France). Horseradish peroxidase (HRP) was from Roche (Mannheim, Germany). Sodium hydrogen phosphate (NaHPO_4_.2H_2_O), potassium dihydrogen phosphate (KH_2_PO_4_), and sodium persulfate (Na_2_S_2_O_8_) were obtained from Aldrich (Leuven, Belgium). All the solutions were prepared with MilliQ water or ultrapure water (Easy Pure UV purification system, Barnested/Thermolyne, Dubuque, IA, USA).

The ^1^H, ^13^C, and ^31^P NMR spectra were recorded at 298 K using a Bruker DRX NMR spectrometer (Rheinstetten, Germany) operating at 400.13, 100.61, and 161.98 MHz, respectively. Chemical shifts were quoted in parts per million (δ) downfield from TMS and were referenced from the residual solvent peaks (CH_2_Cl_2_: 5.32 ppm; CH_2_Cl_2_: 53.24 ppm; CD_2_Cl_2_: 54.84 ppm) [72] or TMS. Spin multiplicities are indicated by the following symbols: s (singlet), d (doublet), t (triplet), and m (multiplet). The coupling constants, J, are reported in Hertz (Hz). High-resolution mass spectrometry analyses were performed in the Laboratory of Mass Spectrometry of the University of Liège on a Bruker DaltonicsSolariX FT-ICR (Bremen, Germany) spectrometer operating at 9.4 T, using the electrospray ionization mode (ESI). Melting points were determined in open glass capillaries using an OSI 9100 Electrothermal digital melting point apparatus Bibby Scientific Limited (Staffordshire, UK) and were uncorrected.

### 3.2. Synthesis of Palladium Complexes

The palladium complexes bearing an iodo ligand (**1**, **4**, and **7**) described in this work were prepared according to a direct oxidative addition method previously reported by our group [43]. The synthesis procedure of these compounds is briefly described in the sections below.

#### 3.2.1. Preparation of Complexes **1**, **4**, and **7**

A 50 mL round-bottom flask equipped with a magnetic stirring bar and capped with a three-way stopcock was charged with Pd(PPh_3_)_4_ (1 eq, 0.288 g, 0.25 mmol) and *para*-substituted iodobenzene (1.05 eq, 0.26 mmol). Dry toluene (10 mL) was added with a syringe. The resulting yellow suspension was stirred for 4 h at room temperature. The suspension was then filtered rapidly by Buchner. The remaining solid was washed with toluene (3 × 10 mL) and *n*-pentane (3 × 10 mL) and dried under high vacuum.

##### Trans-Iodo(4-methoxyphenyl)bis(triphenylphosphine)Palladium C_43_H_37_OIP_2_Pd (**1**)

Yellow powder (0.195 g, 90% yield). ^1^H NMR (400 MHz, CD_2_Cl_2_, ppm): δ 7.55–7.51 (m, 12H, *PPh_3_* CH*_arm_*); 7.39–7.36 (m, 6H, *PPh_3_* CH*_arm_*); 7.31–7.27 (m, 12H, *PPh_3_* CH*_arm_*); 6.46–6.44 (d, *J* = 8 Hz, 2H, CH*_arm_*); 5.94–5.92 (d, *J* = 8 Hz, 2H, CH*_arm_*); 3.48 (s, 3H). ^13^C {H} (100 MHz, CD_2_Cl_2_, ppm): δ 155.94 (s); 145.94–145.89 (t, *J* = 3 Hz); 135.43–135.33 (t, *J* = 5 Hz); 134.75–134.62 (t, *J* = 6 Hz); 132.34–131.88 (t, *J* = 23 Hz); 129.62(s); 127.53–127.43 (t, *J* = 5 Hz); 114.25(s); 54.97 (s). ^31^P {H} (162 MHz, CD_2_Cl_2_, ppm): δ 22.77. HRMS (ESI+): *m*/*z* [(M − I)^+^], calcd. for *C_43_H_37_OP_2_Pd^+^* 737.1208; obsd. 737.1344. m.p.: 172–175 °C.

##### Trans-Iodo(4-acethoxyphenyl)bis(triphenylphosphine)Palladium C_44_H_37_O_2_IP_2_Pd (**4**)

Light yellow powder (0.212 g, 95% yield). ^1^H NMR (400 MHz, CD_2_Cl_2_, ppm): δ 7.54–7.50 (m, 12H, *PPh_3_* CH*_arm_*); 7.40–7.30 (m, 18H, *PPh_3_* CH*_arm_*); 6.62–6.60 (d, *J* = 8 Hz, 2H, CH*_arm_*); 6.07–6.06 (d, *J* = 4 Hz, 2H, CH*_arm_*); 2.14 (s, 3H). ^13^C {H} (100 MHz, CD_2_Cl_2_, ppm): δ 168.77 (s); 154.08–154.03 (t, *J* = 3 Hz); 146.95 (s); 135.54–135.43 (t, *J* = 5.5 Hz); 134.69–134.57 (t, *J* = 6 Hz); 132.06–131.59 (t, *J* = 23 Hz); 129.65(s); 127.66–127.56 (t, *J* = 5 Hz); 120.58(s); 20.73 (s). ^31^P {H} (162 MHz, CD_2_Cl_2_, ppm): δ 22.47. HRMS (ESI+): *m*/*z* [(M − I)^+^], calcd. for *C_44_H_37_O_2_P_2_Pd^+^* 765.1303; obsd. 765.1268. m.p.: 170–172 °C.

##### Trans-Iodo(phenyl)bis(triphenylphosphine)Palladium C_43_H_35_IP_2_Pd (**7**)

Yellow powder (0.171 g, 82% yield). ^1^H NMR (400 MHz, CDCl_3_, ppm): δ 7.56–7.52 (m, 12H, *PPh_3_* CH*_arm_* ); 7.36–7.33 (m, 6H, *PPh_3_* CH*_arm_*); 7.28–7.24 (m, 12H, *PPh_3_* CH*_arm_*); 6.64–6.62 (d, *J* = 8 Hz, 2H, CH*_arm_*); 6.38–6.34 (t, *J* = 8 Hz, 1H, CH*_arm_*); 6.26–6.23 (t, *J* = 6 Hz, 2H, CH*_arm_*). ^13^C {H} (100 MHz, CDCl_3_, ppm): δ 159.25–159.21 (t, *J* = 2 Hz); 136.17–136.07 (t, *J* = 5 Hz); 135.06–134.94 (t, *J* = 6 Hz); 132.53–132.07 (t, *J* = 23 Hz); 129.82 (s); 127.94–127.84 (t, *J* = 5 Hz); 121.97(s). ^31^P {H} (162 MHz, CDCl_3_, ppm): δ 22.30. HRMS (ESI+): *m*/*z* [(M − I)^+^], calcd. for *C_42_H_35_P_2_Pd^+^* 834.0294; obsd. 834.0298. m.p.: 195–197 °C.

#### 3.2.2. Preparation of Complexes **2**, **3**, **5**, **6**, **8** and **9**

A total of 100 mg of complexes **1**, **4**, or **7** were dissolved in CH_2_Cl_2_ (30 mL) and charged in a bulb. Then, 50 mL of saturated solution of KBr or KCl were added, and the resulting biphasic system was stirred vigorously at room temperature. After the extraction of the organic phase, this step was repeated until the halogen exchange was completed, as monitored by ^31^P NMR spectroscopy. After completion of the reaction, the organic layer was dried using anhydrous MgSO_4_. The drying agent was eliminated by filtration and the solvent was removed in vacuo. The light-yellow powder was further dried under high vacuum.

##### Trans-Bromo(4-methoxyphenyl)bis(triphenylphosphine)Palladium C_43_H_37_OBrP_2_Pd (**2**)

Light yellow powder (0.188 g, 92% yield). ^1^H NMR (250 MHz, CDCl_3_, ppm): δ 7.58–7.50 (m, 12H, *PPh_3_* CH*_arm_*); 7.38–7.25 (m, 18H, *PPh_3_* CH*_arm_*); 6.47–6.44 (d, *J* = 7.5 Hz, 2H, CH*_arm_*); 5.98–5.95 (d, *J* = 7.5 Hz, 2H, CH*_arm_*); 3.53 (s, 3H). ^13^C {H} (62.8 MHz, CDCl_3_, ppm): δ 156.33 (s); 144.02–143.90 (t, *J* = 3.8 Hz); 136.29–133.12 (t, *J* = 5.4 Hz); 135.17–134.97 (t, *J* = 6.3 Hz); 132.25–131.53 (t, *J* = 22.6 Hz); 130.02(s); 128.21–128.06 (t, *J* = 4.7 Hz); 114.72(s); 55.78 (s). ^31^P {H} (162 MHz, CD_2_Cl_2_, ppm): δ 23.41. HRMS (ESI+): *m*/*z* [(M − Br)^+^], calcd. for *C_43_H_37_OP_2_Pd^+^* 737.1354; obsd. 737.1340. m.p.: 178–181 °C (Litt: 180–183 °C [73])

##### Trans-Chloro(4-methoxyphenyl) bis(triphenylphosphine)Palladium C_43_H_37_OClP_2_Pd (**3**)

Light brown powder (0.185 g, 96% yield). ^1^H NMR (400 MHz, CD_2_Cl_2_, ppm): δ 7.54–7.49 (m, 12H, *PPh_3_* CH*_arm_*); 7.40–7.37 (m, 6H, *PPh_3_* CH*_arm_*); 7.31–7.28 (m, 12H, *PPh_3_* CH*_arm_*); 6.47–6.45 (d, *J* = 8 Hz, 2H, CH*_arm_*); 5.94–5.92 (d, *J* = 8 Hz, 2H, CH*_arm_*); 3.49 (s, 3H). ^13^C {H} (100 MHz, CD_2_Cl_2_, ppm): δ 156.93 (s); 142.13–142.06 (t, *J* = 3.5 Hz); 136.89–136.78 (t, *J* = 6 Hz); 135.53–137.41 (t, *J* = 6 Hz); 132.42–131.92 (t, *J* = 25 Hz); 130.61(s); 128.72–128.62 (t, *J* = 5 Hz); 115.05(s); 56.01 (s). ^31^P {H} (162 MHz, CD_2_Cl_2_, ppm): δ 23.54. HRMS (ESI+): *m*/*z* [(M − Cl)^+^], calcd. for *C_43_H_37_OP_2_Pd^+^* 737.1354; obsd. 737.1332. m.p.: 163–167 °C.

##### Trans-Bromo(4-acethoxyphenyl)bis(triphenylphosphine)Palladium C_44_H_37_O_2_BrP_2_Pd (**5**)

Light yellow powder (0.186 g, 88% yield). ^1^H NMR (400 MHz, CD_2_Cl_2_, ppm): δ 7.53–7.49 (m, 12H, *PPh_3_* CH*_arm_*); 7.42–7.36 (m, 6H, *PPh_3_* CH*_arm_*); 7.35–7.31 (m, 12H, *PPh_3_* CH*_arm_*); 6.66–6.64 (d, *J* = 8 Hz, 2H, CH*_arm_*); 6.09–6.07 (d, *J* = 8 Hz, 2H, CH*_arm_*); 2.15 (s, 3H). ^13^C {H} (100 MHz, CD_2_Cl_2_, ppm): δ 168.83 (s); 151.25–151.18 (t, *J* = 4 Hz); 146.94 (s); 135.79–135.68 (t, *J* = 5.5 Hz); 134.54–134.42 (t, *J* = 6 Hz); 131.37–130.92 (t, *J* = 22.5 Hz); 129.70(s); 127.77–127.67 (t, *J* = 5 Hz); 120.55(s); 20.72 (s). ^31^P {H} (162 MHz, CD_2_Cl_2_, ppm): δ 22.47. HRMS (ESI+): *m*/*z* [(M − Br)^+^], calcd. for *C_44_H_37_O_2_P_2_Pd^+^* 765.1303; obsd. 765.1290. m.p.: 168–171 °C.

##### Trans-Chloro(4-acethoxyphenyl)bis(triphenylphosphine)Palladium C_44_H_37_O_2_ClP_2_Pd (**6**)

Yellow powder (0.186 g, 93% yield). ^1^H NMR (400 MHz, CD_2_Cl_2_, ppm): δ 7.53–7.49 (m, 12H, *PPh_3_* CH*_arm_*); 7.42–7.39 (m, 6H, *PPh_3_* CH*_arm_*); 7.35–7.31 (m, 12H, *PPh_3_* CH*_arm_*); 6.66–6.64 (d, *J* = 8 Hz, 2H, CH*_arm_*); 6.09–6.07 (d, *J* = 8 Hz, 2H, CH*_arm_*); 2.15 (s, 3H). ^13^C {H} (100 MHz, CD_2_Cl_2_, ppm): δ 169.86 (s); 150.37–150.28 (t, *J* = 4.5 Hz); 147.90 (s); 137.00–136.90 (t, *J* = 5 Hz); 135.45–135.33 (t, *J* = 6 Hz); 132.06–131.61 (t, *J* = 22.5 Hz); 130.73(s); 128.84–128.74 (t, *J* = 5 Hz); 121.43(s); 21.72 (s). ^31^P {H} (162 MHz, CD_2_Cl_2_, ppm): δ 22.54. HRMS (ESI+): *m*/*z* [(M − Cl)^+^], calcd. for *C_44_H_37_O_2_P_2_Pd^+^* 765.1303; obsd. 765.1332. m.p.: 174–176 °C (Litt: 172–178 °C [73])

##### Trans-Bromo(phenyl)bis(triphenylphosphine)Palladium C_42_H_35_BrP_2_Pd (**8**)

Orange powder (0.177 g, 90% yield). ^1^H NMR (400 MHz, CDCl_3_, ppm): δ 7.55–7.51 (m, 12H, *PPh_3_* CH*_arm_* ); 7.37–7.33 (m, 6H, *PPh_3_* CH*_arm_*); 7.29–7.25 (m, 12H, *PPh_3_* CH*_arm_*); 6.66–6.64 (d, *J* = 8 Hz, 2H, CH*_arm_*); 6.40–6.36 (t, *J* = 4 Hz, 1H, CH*_arm_*); 6.27–6.23 (t, *J* = 4 Hz, 2H, CH*_arm_*). ^13^C {H} (100 MHz, CDCl_3_, ppm): δ 156.26–156.19 (t, *J* = 3.5 Hz); 136.40–136.30 (t, *J* = 5 Hz); 134.92–137.79 (t, *J* = 6.5 Hz); 131.86–131.41 (t, *J* = 22.5 Hz); 126.83 (s); 128.02–127.92 (t, *J* = 5 Hz); 127.83 (s); 121.89(s). ^31^P {H} (162 MHz, CDCl_3_, ppm): δ 23.88. HRMS (ESI+): *m*/*z* [(M − Br)^+^], calcd. for *C_42_H_35_P_2_Pd^+^* 834.0294; obsd. 834.0308. m.p.: 217–219 °C (Litt: 216–220 °C [73]).

##### Trans-Chloro(phenyl)bis(triphenylphosphine)Palladium C_42_H_35_ClP_2_Pd (**9**)

Yellow powder (0.173 g, 93% yield). ^1^H NMR (250 MHz, CDCl_3_) δ 7.49–7.44 (m, 12H, *PPh_3_* CH*_arm_*); 7.35–7.19 (m, 18H, *PPh_3_* CH*_arm_*); 6.62–6.59 (d, *J* = 7.5 Hz, 2H, CH*_arm_*); 6.38–6.32 (t, *J* = 7.5 Hz, 1H, CH*_arm_*); 6.23–6.17 (t, *J* = 7.5 Hz, 2H, CH*_arm_*). ^31^P {H} (101 MHz, CDCl_3_, ppm): δ 23.00. HRMS (ESI+): *m*/*z* [(M − Cl)^+^], calcd. for *C_42_H_35_P_2_Pd^+^* 834.0294; obsd. 834.0311. m.p.: 238–238 °C.

### 3.3. X-Ray Crystal Structure Determination

Crystal data were collected by applying the omega and phi scans’ method on a Bruker APPEX II or Smart CCD-1000 Diffractometer (Karlsruhe, Germany) using graphite-monochromated Mo-Kα radiation (λ = 0.71073 Å) from a fine-focus sealed tube source at 100 K. Computing data and reduction was performed using the APPEX II software [74]. The structure was solved using DIRDIF [75] and finally refined by a full-matrix, least-squares method based on F^2^ by SHELXL [76]. An empirical absorption correction was applied using SADABS [77]. All non-hydrogen atoms were anisotropically refined, and the hydrogen atom positions were included in the model by electronic density or were geometrically calculated and refined using a riding model.

The crystal data, data collection, and structure refinement details of complexes **1** and **2** are summarized in Table 1. Crystallographic data have been deposited at the Cambridge Crystallographic Data Centre with the numbers CCDC-2105940 (complex **1**), and CCDC-2105941 (complex **2**). These data can be obtained, free of charge, from CCDC, 12 Union Road, Cambridge, CB2 1EZ, UK (fax: +44 1233 336033; e-mail: deposit@ccdc.cam.ac.uk; internet: http://www.ccdc.cam.ac.uk). 

### 3.4. Free Radicals Scavenging Methods

Radical scavenging capacity, as an indicator of the antioxidant capacity of compounds against 1,1-diphenyl-2-picrylhydrazyl (DPPH) free radicals and 2,2-azinobis (3-ethyl- enzothiazoline-6-sulfonic acid), ABTS•+ radical cations, was determined using a spectrophotometric assay [78]. Antiradical capacity analysis was performed either on pure MeOH for ABTS or the mixture of MeOH/distilled water (90:10 *v*/*v*) for DPPH. For both ABTS and DPPH assays, the stock solutions of complexes were prepared in DMSO. Quercetin was used as an antioxidant standard in the DPPH and ABTS•+ scavenging tests. EC_50_ is defined as the concentration of compound (µM) required to scavenge 50% of ABTS•+ or DPPH radicals. EC_50_ values were estimated using nonlinear regression. A lower EC_50_ value indicates higher antiradical activity. 

#### 3.4.1. ABTS Test

The ABTS assay was based on the method described by Re et al. [79]. For the generation of ABTS•+ radicals, sodium persulfate (2.45 mM) aqueous solution was mixed with ABTS (7 mM) and incubated overnight in the dark to obtain a dark colored solution. The stock solution of ABTS•+ was then diluted by adding pure methanol (100%) to obtain an absorbance of 0.70 (±0.02) at 734 nm at 30 °C. An aliquot of 0.02 mL of tested compound was added to 1.98 mL of ABTS•+, and the decrease in absorbance was monitored at 734 nm after 30 min [80]. During this reaction, the blue green ABTS radical cation is converted back into its colorless neutral form in the presence of the potential antioxidant molecule. A control consisted of 0.02 mL of DMSO in 1.98 mL of ABTS•+ solution. The reducing capacity was determined according to the following formula:

% inhibition = (A _control_ − A _Sample_) × 100/A _Control_ (1)

#### 3.4.2. DPPH Test

The DPPH assay was performed according to the method developed by Brand-Williams et al. slightly modified [81]. A solution of 1 mM DPPH in 90% (*v*/*v*) methanol was stirred for 40 min. The absorbance of the solution was adjusted to 0.650 ± 0.020 at 517 nm using fresh 90% (*v*/*v*) methanol. Then, 0.02 mL of quercetin taken as a standard or sample was mixed with 1.98 mL of DPPH solution and incubated for 30 min in the dark covered with aluminum foil. When reacting with an antioxidant, the DPPH• radical is converted into DPPH, and its color changes from purple to yellow. The antioxidant effect may be easily evaluated by observing the decrease in visible absorption. The absorbance decrease was monitored at 517 nm after 30 min of incubation with a Hewlett Packard 8453 spectrophotometer. The control consisted of 0.02 mL of DMSO in 1.98 mL of DPPH solution. A similar formula (Equation (1)) was applied to determine the DPPH radical scavenging activity.

### 3.5. Antiradical Efficiency

The reaction between potential antioxidants and the oxidized substrate requires a steady state which will depend on several parameters, such as the reaction time. The time needed to reach the steady state, at the concentration corresponding to EC_50_, is more and more frequently used and can be defined as T_EC50_. In brief, T_EC50_ was determined by plotting the recorded absorbance during the period for the EC_50_ concentration for the considered antioxidant compound. The parameter to express antioxidant capacity, called “antiradical efficiency” (AE), was defined as follows:

AE = 1/EC_50_.T_EC50_

The antiradical efficiency of compounds and their classification are considered according to the work of Sanchez-Moreno [69]. The value of the AE determines if the activity of the potential antioxidant is low or high (AE ≤ 1.10^−^^3^ low; 1.10^−^^3^ < AE ≤ 5.10^−^^3^ medium; 5.10^−^^3^ < AE ≤ 10.10^−^^3^ high; and 10.10^−^^3^ < AE very high).

### 3.6. Chemiluminescence Study of Complexes on Enzyme-Catalyzed Oxidation of L012 

To investigate the effect of selected palladium (II)-based complexes on the peroxidase (HRP)-catalyzed oxidation of the chemiluminescent probe (L-012), in the presence of hydrogen peroxide (H_2_O_2_), the chemiluminescence technique was used. Briefly, plate-reader-based luminescence measurements were performed in white 96-well plates using a Fluoroskan Ascent FL microplate reader (Thermo Labsystems, Vantaa, Finland), with the temperature set at 37 °C. To 5 µL of a stock solution of HRP (1 mg/mL) in phosphate buffer (pH = 7.5) per well, we successively added 10 µL of L-012 (aqueous solution, 0.84 mmoL) and 2 µL of tested compound in 163 µL of buffer solution. The reaction was triggered by adding 20 µL hydrogen peroxide (0.19 µM) to reach the final volume of 200 µL. The light emission, produced from oxidation of L-012 by HRP/H_2_O_2_ system, in the presence or absence of the tested palladium complex, was recorded in kinetic mode for 30 min. Inhibition of the emitted light due to tested complexes was associated with higher activity. The following formula was applied to calculate the inhibiting activity of complexes towards the oxidation of L-012 catalyzed by the HRP/H_2_O_2_ system:

% inhibition = (L _control_ − L _Sample_) x 100/L _Control_

### 3.7. Statistical Analysis

Results were expressed as mean values ± standard deviation (SD). All measurements were replicated three times. EC_50_ values were calculated with GraphPad Prism 6.0 (GraphPad Software, San Diego, CA, USA) under the application of the function “log (inhibitor) versus normalized response-variable slope” after converting the concentrations into their decimal logarithm. The results were analyzed using Student’s test and a two-way analysis of variance (ANOVA), and a multiple comparison of all data was performed using Tukey’s multiple comparisons test. *p* < 0.0001 was considered significant.

## 4. Conclusions

Overall, herein we have developed a convenient one-pot water-compatible procedure for the synthesis of the *trans* [PdX(PPh_3_)_2_(4-RC_6_H_4_)] complex. This method is simple and requires a lower quantity of halogen salt to prepare other attached halogen complex homologs. Two novel complexes were synthesized by this new method. Molecular structures obtained by X-ray diffraction for the more stable complexes did not show an isostructural relationship to the *para*-methoxyphenyl ligand moiety of complexes, with the *para*-acetoxyphenyl ligand crystallized with one molecule of dichloromethane used as a solvent. The radical scavenging activity of the complexes was evaluated with ABTS and DPPH models, and also with an enzymatic model using HRP-H_2_O_2_/L-012. Taken together, the complexes (**1**, **2**, and **3**) have shown very good activity in the three models studied. All the tests confirm that the complex bearing the *para*-methoxyphenyl ligand is the best radical scavenger candidate. The kinetic studies also reveal that all the complexes react more quickly and easily with DPPH radicals in an exponential way. The study of antiradical efficiency confirms the need for kinetic parameters before the classification of the efficiency of any molecule being investigated. Although no final evidence is offered in this work to formally identify the mechanism behind the interaction between these palladium complexes and DPPH or ABTS radicals, this study paves the way toward deepening our knowledge of this mechanism. A study is currently underway in our laboratory to identify the effective process of the radical scavenging reaction.

## Figures and Tables

**Table 1:** Crystal data for compounds **1** and **2**.

Crystal Data	1	2
Chemical formula	C_43_H_37_OIP_2_Pd	C_43_H_37_OBrP_2_Pd
Mr	864.96	817.97
Crystal system, space group	Monoclinic, *Ia*	Orthorhombic, *Pbca*
Temperature (K)	100	100
*a* (Å)	11.3164 (7)	11.4818 (11)
*B* (Å)	13.5866 (8)	23.717 (3)
*c* (Å)	23.3413 (16)	26.133 (3)
*β* (°)	94.306 (3)	-
*V* (Å^3^)	3578.6 (4)	7116.3 (13)
*Z*	4	8
Radiation type	Mo-Kα radiation	Mo-Kα radiation
*μ* (mm^−1^)	1.50	1.77
Crystal size (mm)	0.22 × 0.20 × 0.11	0.27 × 0.08 × 0.06
Data collection		
Diffractometer	BRUKER APPEX-II CCD	BRUKER APPEX-II
Absorption correction	Multi-scan BRUKER SADABS2012/1	Multi-scan SADABS2016/2—Bruker AXS area detector scaling and absorption correction
*Tmin, Tmax*	0.765, 0.825	0.693, 0.801
No. of measured, independent and observed *[I > 2σ(I)]* reflections	64342, 8854, 8583	101525, 6743, 5008
*R*_int_	0.056	0.117
(sin θ/λ)max (Å^−1^)	0.667	0.610
Refinement		
R[F2 > 2σ(F2)], wR(F2), S	0.022, 0.048, 1.04	0.041, 0.107, 1.06
No. of reflections	8854	6743
No. of parameters	435	434
No. of restraints	2	-
H-atom treatment	H-atom parameters constrained	H-atom parameters constrained
Δρ_max_, Δρ_min_ (e Å^−3^)	0.56, −0.30	0.64, −1.38

**Table 2:** Selected bond(s)’s distances (Å) and angles (°).

Bond Parameters (Distance (Å) and Angle (°))	Complex 1	Complex 2
Pd–X	2.7009 (3)	2.5120 (5)
P1–Pd1	2.3343 (9)	2.3417 (11)
P2–Pd1	2.3307 (10)	2.3216 (11)
Pd1–C37	2.019 (3)	2.015 (4)
C40–O1	1.380 (5)	1.392 (5)
C43–O1	1.426 (5)	1.438 (5)
C37–Pd1–P2	88.75 (9)	91.33 (11)
C37–Pd1–P1	88.45 (9)	91.93 (11)
P2–Pd1–P1	176.86 (3)	173.39 (4)
C37–Pd1–X	179.27 (11)	177.18 (10)
P2–Pd1–X	91.18 (3)	86.49 (3)
P1–Pd1–X	91.60 (3)	90.06 (3)
C40–O1–C43	115.7 (3)	117.1 (3)

**Table 3:** EC_50_ values of tested compounds in comparison to quercetin (**Qrcn**), taken as the antioxidant reference.

Compounds	EC_50_ (µM)	
	ABTS	DPPH
**Qrcn**	5.56 ± 0.97	5.79 ± 1.00
**1**	5.78 ± 0.98	1.14 ± 0.90
**2**	7.01 ± 0.98	7.09 ± 0.94
**3**	11.12 ± 0.94	1.90 ± 0.87
**4**	10.89 ± 0.95	1840.77 ± 0.44
**5**	135.83 ± 0.25	15.45 ± 0.96
**6**	45.81 ± 0.95	14.32 ± 0.92
**7**	6.14 ± 0.94	45.08 ± 0.95
**8**	14.32 ± 0.92	6.64 ± 0.91
**9**	10.35 ± 0.64	7.33 ± 0.97

**Table 4:** Classification, EC_50_, T_EC50_, and AE values for complexes **1**, **2**, **3**, **8**, and **9**, compared to quercetin.

Compounds	EC_50_ (g Antioxidant/kg DPPH°)	T_EC50_ (min)	AE (×10^−3^)	Classification
**1**	49.31 ± 39	11	1.8437	Medium
**2**	2898.99 ± 39	16	0.2155	Low
**3**	73.49 ± 38	63	0.2160	Low
**4**	82,194.89 ± 33	78	0.0002	Low
**5**	653.57 ± 20	58	0.0264	Low
**6**	573.94 ± 40	53	0.0328	Low
**7**	1882.11 ± 37	66	0.0081	Low
**8**	260.96 ± 40	40	0.0955	Low
**9**	271.97 ± 36	36	0.1019	Low
**Qrcn**	87.98 ± 15	61	0.1873	Low

**Table 5:** EC_50_ value of tested compounds in chemiluminescence assay.

Compounds	EC_50_ (µM)
**Qrcn**	7.06 ± 2.56
**1**	3.56 ± 1.87
**2**	1.48 ± 0.71
**3**	5.80 ± 2.60
**4**	11.29 ± 3.30
**5**	5.28 ± 2.30
**6**	6.46 ± 2.28
**7**	66.99 ± 20.11
**8**	33.34 ± 11.71
**9**	39.17 ± 17.04

## Data Availability

Data is contained within the article.

## Supplementary Materials

The following supporting information can be downloaded at: https://www.mdpi.com/article/10.3390/molecules30051122/s1, Figure S1. ^1^H NMR spectrum of 1. Figure S2. ^13^C NMR spectrum of 1. Figure S3. ^31^P NMR spectrum of 1. Figure S4. ^1^H NMR spectrum of 2. Figure S5. ^13^C NMR spectrum of 2. Figure S6. ^31^P NMR spectrum of 2. Figure S7. ^1^H NMR spectrum of 3. Figure S8. ^13^C NMR spectrum of 3. Figure S9. ^31^P NMR spectrum of 3. Figure S10. ^1^H NMR spectrum of 4. Figure S11. ^13^C NMR spectrum of 4. Figure S12. ^31^P NMR spectrum of 4. Figure S13. ^1^H NMR spectrum of 5 Figure S14. ^13^C NMR spectrum of 5. Figure S15. ^31^P NMR spectrum of 5. Figure S16. ^1^H NMR spectrum of 6. Figure S17. ^13^C NMR spectrum of 6. Figure S18. ^31^P NMR spectrum of 6. Figure S19. ^1^H NMR spectrum of 7. Figure S20. ^13^C NMR spectrum of 7. Figure S21. ^31^P NMR spectrum of 7. Figure S22. ^1^H NMR spectrum of 8. Figure S23. ^13^C NMR spectrum of 8. Figure S24. ^31^P NMR spectrum of 8.n. Figure S25. ^1^H NMR spectrum of 9. Figure S26. ^13^C NMR spectrum of 9. Figure S27. ^31^P NMR spectrum of 9. Figure S28. ORTEP representation of 1 with thermal ellipsoids drawn at the 50% probability level showing I—H interactions. Figure S29. ORTEP representation of 2 with thermal ellipsoids drawn at the 50% probability level showing O—H interactions. Figure S30. ORTEP representation of byproduct isolated from complex 3 solution resulting from self-decomposition/rearrangement reaction. Table S1. Crystal data for compounds 1 and 2.
