# Supplementary material for: Free Radical Scavenging Activity and Inhibition of Enzyme-Catalyzed Oxidation by trans-aryl-Palladium Complexes
Source: Molecules. 2025 Feb 28;30(5):1122. doi: 10.3390/molecules30051122 (PMC11901561; doi:10.3390/molecules30051122)
Supplement: Supplementary file 1 [file molecules-30-01122-s001.zip › molecules-3473987-supplementary.pdf]

# EXPERIMENTAL SECTION

# Free radical scavenging activity and inhibition of enzyme-catalyzed oxidation by *trans*-aryl-palladium complexes

Koffi Sénam Etsè[a,b], Mohamed Anouar Harrad[c,d], Kodjo Djidjolé Etsè[e], Guillermo Zaragoza[f], Albert Demonceau[a], Ange Mouithys-Mickalad[g]

[a]*Laboratory of Macromolecular Chemistry and Organic Catalysis, Departement of Chemistry, University of Liège, Sart-Tilman (B.6a), 4000 Liège, Belgium*

[b]*Laboratory of Medicinal Chemistry, Center for Interdisciplinary Research on Medicines (CIRM), University of Liège, Quartier Hôpital B36 Av. Hippocrate 15 Liège B-4000, Belgium*

[c]*Environmental, Ecological, and Agro-Industrial Engineering Laboratory, Sultan Moulay Slimane University, P.O Box 523, Beni-mellal 23000, Morocco*

[d]*Regional centre for Education training and formation, CRMEF 40000 Marrakech-Safi, Morocco*

[e]*Laboratoire de Physiologie et Biotechnologie Végétales (LPBV), Facultédes Sciences (FDS), Universitéde Lomé(UL), Lomé, Togo*

[f]*Unidade de Difracción de Raios X, Universidade de Santiago de Compostela, Edificio CACTUS,Campus Vida, 15782 Santiago de Compostela, Spain*

[g]*Center for Oxygen, Research and Development (CORD), Veterinary Clinic, Quartier Vallee 2, Avenue de Cureghem 5. University of Liège, Sart-Tilman (B.6a), 4000 Liège, Belgium*

## 1. General Information

Analytical-grade methanol and ethyl acetate (Chem-Lab, Zedelgem, Belgium) were used in the extraction procedure. Sodium chloride (NaCl), potassium chloride (KCl), potassium bromide (KBr), ammonium acetate, potassium hydroxide, sodium hydroxide, acetic acid, ethanol, hydrogen peroxide ($H_2O_2$), dimethyl sulfoxide (DMSO), were all purchased from Merck (VWRI, Leuven, Belgium). $Pd(PPh_3)_4$, *para*-iodomethoxyphenyl, *para*-acetoxyphenyl, iodobenzene, toluene were obtained from Aldrich (Belgium). ABTS 2,2- azinobis (3-ethyl-enzothiazoline-6-sulfonic acid) and DPPH (1,1-diphenyl-2-picrylhydrazyl) were purchased from Aldrich (Belgium). Quercetin (3,3',4',5,7-pentahydroxy-2-phenylchromen-4-one) is from ChromaDex (LGC Standard, France). Horseradish peroxidase (HRP) was from Roche (Germany). Sodium hydrogen phosphate ($NaHPO_4.2H_2O$), potassium dihydrogen phosphate ($KH_2PO_4$) and sodium persulfate ($Na_2S_2O_8$) were obtained from Aldrich (Belgium). All the solutions were prepared with MilliQ water or ultrapure water (Easy Pure UV purification system, Barnested/Thermolyne, Dubuque, USA).

## 2. Synthesis and characterization of Palladium complexes

The palladium complexes bearing iodo ligand (**1**, **4** and **7)** described in this work were prepared according to direct oxidative addition method previously reported by our group [1]. The synthesis procedure of these compounds are briefly described in the sections below.

### *2.1. Preparation of complexes **1**, **4** and **7**.*

A 50 mL round-bottom flask equipped with a magnetic stirring bar and capped with a three-way stopcock was charged with $Pd(PPh_3)_4$ (1 eq, 0.288 g, 0.25 mmol) and an *para* substituted iodobenzene (1.05 eq, 0.26 mmol). Dry toluene (10 mL) was added with a syringe. The resulting yellow suspension was stirred for 4 h at room temperature. The suspension was then filtered rapidly by Buchner. The remaining solid was washed with toluene (3 × 10 mL) and *n*-pentane (3 × 10 mL) and dried under high vacuum.

#### *2.2.1. Trans-Iodo(4-methoxyphenyl)bis(triphenylphosphine)palladium $C_{43}H_{37}OIP_2Pd$ (**1**).*

Yellow powder (0,195 g, 90% yield). $^1H$ NMR (400 MHz, $CD_2Cl_2$, ppm): δ 7.55-7.51 (m, 12H, *PPh₃* $CH_{arm}$); 7.39-7.36 (m, 6H, *PPh₃* $CH_{arm}$); 7.31-7.27 (m, 12H, *PPh₃* $CH_{arm}$); 6.46-6.44 (d, *J* = 8 Hz, 2H, $CH_{arm}$); 5.94-5.92 (d, *J* = 8 Hz, 2H, $CH_{arm}$); 3.48 (s, 3H). $^{13}C$ {H} (100 MHz,

$CD_2Cl_2$, ppm): δ 155.94 (s); 145.94-145.89 (t, $J$ = 3 Hz); 135.43-135.33 (t, $J$ = 5 Hz); 134.75-134.62 (t, $J$ = 6 Hz); 132.34-131.88 (t, $J$ = 23 Hz); 129.62(s); 127.53-127.43 (t, $J$ = 5 Hz); 114.25(s) ; 54.97 (s). $^{31}$P {H} (162 MHz, $CD_2Cl_2$, ppm): δ 22.77. HRMS (ESI+): m/z [(M - I)$^+$], calcd. for $C_{43}H_{37}OP_2Pd^+$ 737.1208; obsd. 737.1344.

*2.2.2. Trans-Iodo(4-acethoxyphenyl)bis(triphenylphosphine)palladium $C_{44}H_{37}O_2IP_2Pd$ (**4**).*

Light yellow powder (0,212 g, 95% yield). $^1$H NMR (400 MHz, $CD_2Cl_2$, ppm): δ 7.54-7.50 (m, 12H, *PPh3* $CH_{arm}$ ); 7.40-7.30 (m, 18H, *PPh3* $CH_{arm}$); 6.62-6.60 (d, $J$ = 8 Hz, 2H, $CH_{arm}$); 6.07-6.06 (d, $J$ = 4 Hz, 2H, $CH_{arm}$); 2.14 (s, 3H). $^{13}$C {H} (100 MHz, $CD_2Cl_2$, ppm): δ 168.77 (s); 154.08-154.03 (t, $J$ = 3 Hz); 146.95 (s) ; 135.54-135.43 (t, $J$ = 5.5 Hz) ; 134.69-134.57 (t, $J$ = 6 Hz); 132.06-131.59 (t, $J$ = 23 Hz) ; 129.65(s) ; 127.66-127.56 (t, $J$ = 5 Hz) ; 120.58(s) ; 20.73 (s). $^{31}$P {H} (162 MHz, $CD_2Cl_2$, ppm): δ 22.47. HRMS (ESI+): m/z [(M - I)$^+$], calcd. for $C_{44}H_{37}O_2P_2Pd^+$ 765.1303; obsd. 765.1268.

*2.2.3. Trans-iodo(phenyl)bis(triphenylphosphine)palladium $C_{43}H_{35}IP_2Pd$ (**7**).*

Yellow powder (0,171 g, 82% yield). $^1$H NMR (400 MHz, $CDCl_3$, ppm): δ 7.56-7.52 (m, 12H, *PPh3* $CH_{arm}$ ); 7.36-7.33 (m, 6H, *PPh3* $CH_{arm}$); 7.28-7.24 (m, 12H, *PPh3* $CH_{arm}$); 6.64-6.62 (d, $J$ = 8 Hz, 2H, $CH_{arm}$); 6.38-6.34 (t, $J$ = 8 Hz, 1H, $CH_{arm}$); 6.26-6.23 (t, $J$ = 6 Hz, 2H, $CH_{arm}$). $^{13}$C {H} (100 MHz, $CDCl_3$, ppm): δ 159.25-159.21 (t, $J$ = 2 Hz); 136.17-136.07 (t, $J$ = 5 Hz); 135.06-134.94 (t, $J$ = 6 Hz); 132.53-132.07 (t, $J$ = 23 Hz); 129.82 (s) ; 127.94-127.84 (t, $J$ = 5 Hz) ; 121.97(s). $^{31}$P {H} (162 MHz, $CDCl_3$, ppm): δ 22.30. HRMS (ESI+): m/z [(M - I)$^+$], calcd. for $C_{42}H_{35}P_2Pd^+$ 834.0294; obsd. 834.0298

*2.3 Preparation of complexes **2**, **3**, **5**, **6**, **8** and **9**.*

Complex **1**, **4** or **7** (100 mg) was dissolved in $CH_2Cl_2$ (30 mL) and charged in bulb. 50 mL of saturated solution of KBr or KCl was added and the resulting biphasic system was stirred vigorously at room temperature. After extraction of the organic phase, this step was repeated until halogen exchange was complete as monitored by $^{31}$P NMR spectroscopy. After completion of the reaction, the organic layer was dried using anhydrous $MgSO_4$. The drying agent was eliminated by filtration and the solvent removed in vacuo. The light-yellow powder was further dried under high vacuum.

*2.3.1. trans-Bromo(4-methoxyphenyl)bis(triphenylphosphine)palladium $C_{43}H_{37}OBrP_2Pd$ (**2**).*

Light yellow powder (0,188 g, 92% yield). $^{1}$H NMR (250 MHz, $CDCl_3$, ppm): δ 7.58-7.50 (m, 12H, *$PPh_3$* $CH_{arm}$); 7.38-7.25 (m, 18H, *$PPh_3$* $CH_{arm}$); 6.47-6.44 (d, *J* = 7.5 Hz, 2H, $CH_{arm}$); 5.98-5.95 (d, *J* = 7.5 Hz, 2H, $CH_{arm}$); 3.53 (s, 3H). $^{13}$C {H} (62.8 MHz, $CDCl_3$, ppm): δ 156.33 (s); 144.02-143.90 (t, *J* = 3.8 Hz); 136.29-133.12 (t, *J* = 5.4 Hz); 135.17-134.97 (t, *J* = 6.3 Hz); 132.25-131.53 (t, *J* = 22.6 Hz); 130.02(s); 128.21-128.06 (t, *J* = 4.7 Hz); 114.72(s) ; 55.78 (s). $^{31}$P {H} (162 MHz, $CD_2Cl_2$, ppm): δ 23.41. HRMS (ESI+): m/z [(M - Br)$^+$], calcd. for $C_{43}H_{37}OP_2Pd^+$ 737.1354; obsd. 737.1340.

*2.3.2. trans-Chloro(4-methoxyphenyl) bis(triphenylphosphine)palladium $C_{43}H_{37}OClP_2Pd$ (**3**).*

Light brown powder (0,185 g, 96% yield). $^{1}$H NMR (400 MHz, $CD_2Cl_2$, ppm): δ 7.54-7.49 (m, 12H, *$PPh_3$* $CH_{arm}$); 7.40-7.37 (m, 6H, *$PPh_3$* $CH_{arm}$); 7.31-7.28 (m, 12H, *$PPh_3$* $CH_{arm}$); 6.47-6.45 (d, *J* = 8 Hz, 2H, $CH_{arm}$); 5.94-5.92 (d, *J* = 8 Hz, 2H, $CH_{arm}$); 3.49 (s, 3H). $^{13}$C {H} (100 MHz, $CD_2Cl_2$, ppm): δ 156.93 (s); 142.13-142.06 (t, *J* = 3.5 Hz); 136.89-136.78 (t, *J* = 6 Hz); 135.53-137.41 (t, *J* = 6 Hz); 132.42-131.92 (t, *J* = 25 Hz); 130.61(s) ; 128.72-128.62 (t, *J* = 5 Hz) ; 115.05(s) ; 56.01 (s). $^{31}$P {H} (162 MHz, $CD_2Cl_2$, ppm): δ 23.54. HRMS (ESI+): m/z [(M - Cl)$^+$], calcd. for $C_{43}H_{37}OP_2Pd^+$ 737.1354; obsd. 737.1332.

*2.3.3. trans-Bromo(4-acethoxyphenyl)bis(triphenylphosphine)palladium $C_{44}H_{37}O_2BrP_2Pd$ (**5**).*

Light yellow powder (0,186 g, 88% yield). $^{1}$H NMR (400 MHz, $CD_2Cl_2$, ppm): δ 7.53-7.49 (m, 12H, *$PPh_3$* $CH_{arm}$); 7.42-7.36 (m, 6H, *$PPh_3$* $CH_{arm}$); 7.35-7.31 (m, 12H, *$PPh_3$* $CH_{arm}$); 6.66-6.64 (d, *J* = 8 Hz, 2H, $CH_{arm}$); 6.09-6.07 (d, *J* = 8 Hz, 2H, $CH_{arm}$); 2.15 (s, 3H). $^{13}$C {H} (100 MHz, $CD_2Cl_2$, ppm): δ 168.83 (s); 151.25-151.18 (t, *J* = 4 Hz); 146.94 (s); 135.79-135.68 (t, *J* = 5.5 Hz) ; 134.54-134.42 (t, *J* = 6 Hz); 131.37-130.92 (t, *J* = 22.5 Hz); 129.70(s) ; 127.77-127.67 (t, *J* = 5 Hz); 120.55(s) ; 20.72 (s). $^{31}$P {H} (162 MHz, $CD_2Cl_2$, ppm): δ 22.47. HRMS (ESI+): m/z [(M - Br)$^+$], calcd. for $C_{44}H_{37}O_2P_2Pd^+$ 765.1303; obsd. 765.1290.

*2.3.4. trans-Chloro(4-acethoxyphenyl)bis(triphenylphosphine)palladium $C_{44}H_{37}O_2ClP_2Pd$ (**6**).*

Yellow powder (0,186 g, 93% yield). $^{1}$H NMR (400 MHz, $CD_2Cl_2$, ppm): δ 7.53-7.49 (m, 12H, *$PPh_3$* $CH_{arm}$); 7.42-7.39 (m, 6H, *$PPh_3$* $CH_{arm}$); 7.35-7.31 (m, 12H, *$PPh_3$* $CH_{arm}$); 6.66-6.64 (d, *J* = 8 Hz, 2H, $CH_{arm}$); 6.09-6.07 (d, *J* = 8 Hz, 2H, $CH_{arm}$); 2.15 (s, 3H). $^{13}$C {H} (100 MHz, $CD_2Cl_2$, ppm): δ 169.86 (s); 150.37-150.28 (t, *J* = 4.5 Hz); 147.90 (s) ; 137.00-136.90 (t, *J* = 5 Hz) ; 135.45-135.33 (t, *J* = 6 Hz); 132.06-131.61 (t, *J* = 22.5 Hz); 130.73(s); 128.84-128.74 (t, *J* = 5 Hz) ; 121.43(s) ; 21.72 (s). $^{31}$P {H} (162 MHz, $CD_2Cl_2$, ppm): δ 22.54. HRMS (ESI+): m/z [(M - Cl)$^+$], calcd. for $C_{44}H_{37}O_2P_2Pd^+$ 765.1303; obsd. 765.1332.

*2.3.5. Trans-bromo(phenyl)bis(triphenylphosphine)palladium $C_{42}H_{35}BrP_2Pd$ (**8**).*

Orange powder (0,177 g, 90% yield). $^1$H NMR (400 MHz, $CDCl_3$, ppm): δ 7.55-7.51 (m, 12H, *PPh₃* CH$_{arm}$ ); 7.37-7.33 (m, 6H, *PPh₃* CH$_{arm}$); 7.29-7.25 (m, 12H, *PPh₃* CH$_{arm}$); 6.66-6.64 (d, *J* = 8 Hz, 2H, CH$_{arm}$); 6.40-6.36 (t, *J* = 4 Hz, 1H, CH$_{arm}$); 6.27-6.23 (t, *J* = 4 Hz, 2H, CH$_{arm}$). $^{13}$C {H} (100 MHz, $CDCl_3$, ppm): δ 156.26-156.19 (t, *J* = 3.5 Hz); 136.40-136.30 (t, *J* = 5 Hz); 134.92-137.79 (t, *J* = 6 .5 Hz); 131.86-131.41 (t, *J* = 22.5 Hz); 126.83 (s); 128.02-127.92 (t, *J* = 5 Hz) ; 127.83 (s) ; 121.89(s). $^{31}$P {H} (162 MHz, $CDCl_3$, ppm): δ 23.88. HRMS (ESI+): m/z [(M - Br)$^+$], calcd. for $C_{42}H_{35}P_2Pd^+$ 834.0294; obsd. 834.0308.

*2.3.6. Trans-chloro(phenyl)bis(triphenylphosphine)palladium $C_{42}H_{35}ClP_2Pd$ (**9**).*

Yellow powder (0,173 g, 93% yield). $^1$H NMR (250 MHz, $CDCl_3$) δ 7.49 - 7.44 (m, 12H, *PPh₃* CH$_{arm}$); 7.35-7.19 (m, 18H, *PPh₃* CH$_{arm}$); 6.62 - 6.59 (d, *J* = 7.5 Hz, 2H, CH$_{arm}$); 6.38 - 6.32 (t, *J* = 7.5 Hz, 1H, CH$_{arm}$); 6.23 - 6.17 (t, *J* = 7.5 Hz, 2H, CH$_{arm}$). $^{31}$P {H} (101 MHz, $CDCl_3$, ppm): δ 23.00. HRMS (ESI+): m/z [(M - Cl)$^+$], calcd. for $C_{42}H_{35}P_2Pd^+$ 834.0294; obsd. 834.0311.

## 3. NMR spectra of complexes 1 to 9

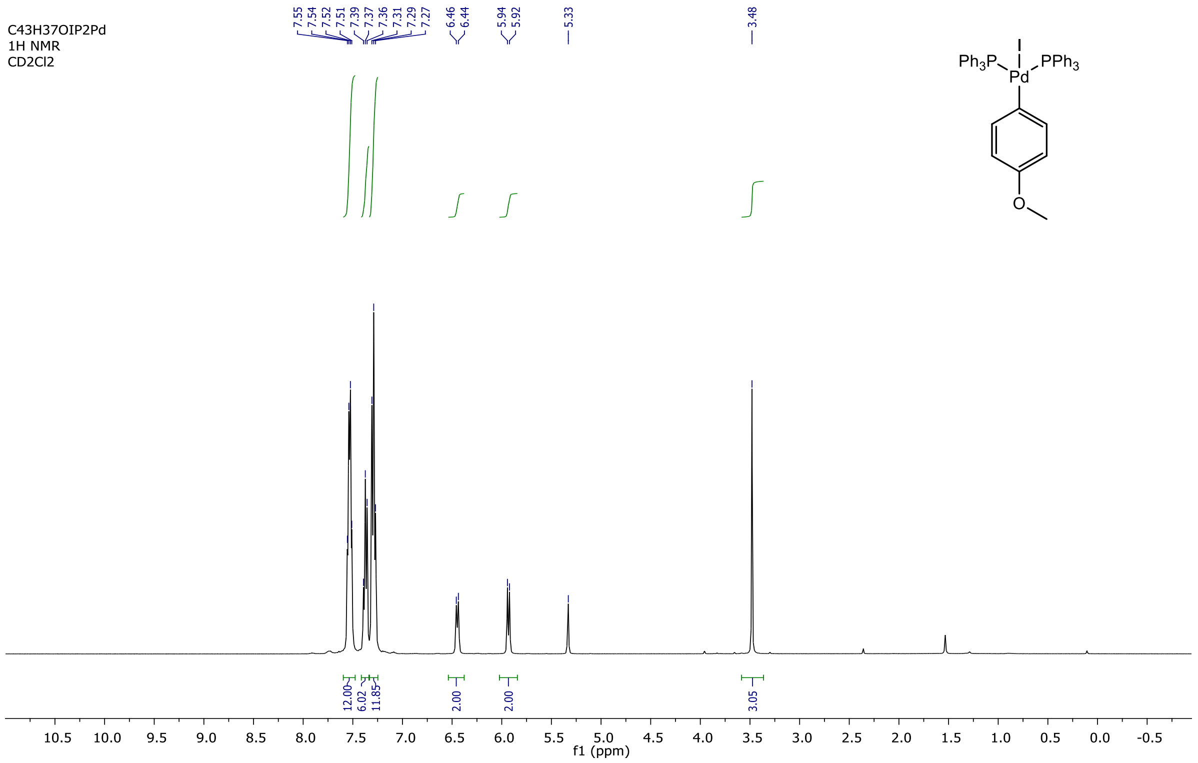

**Figure S1.** ¹H NMR spectrum of **1**.

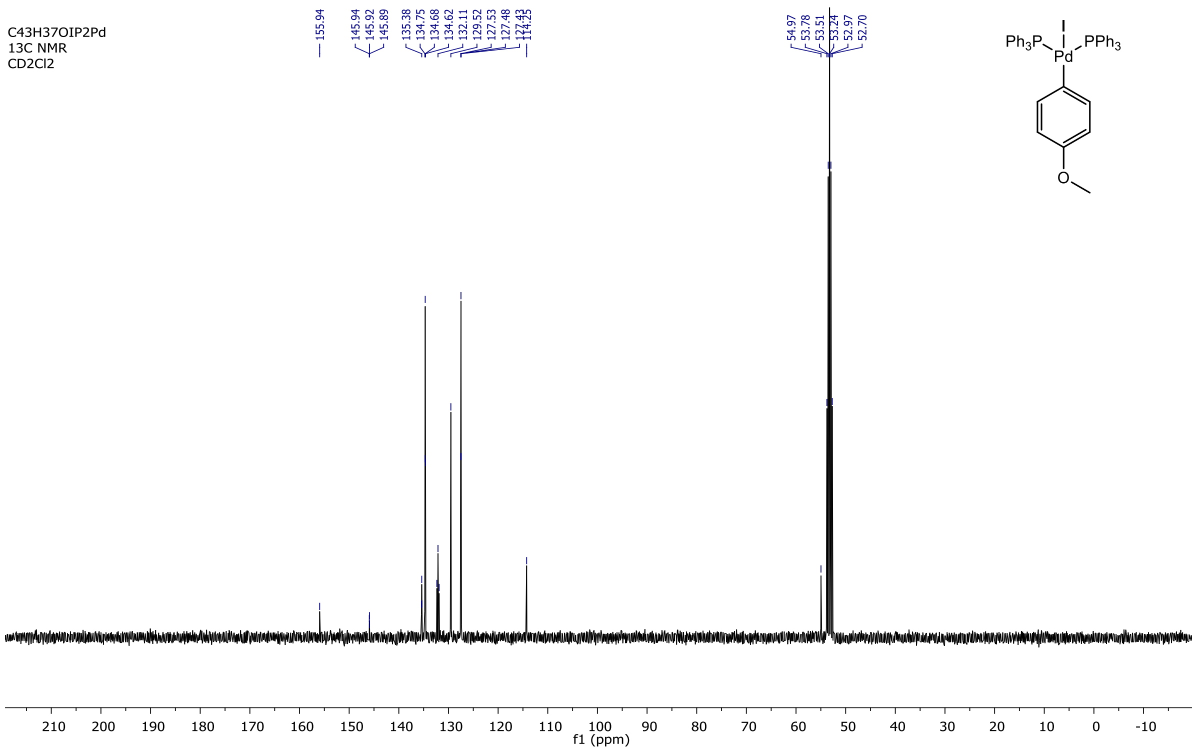


**Figure S2.** ¹³C NMR spectrum of **1**.

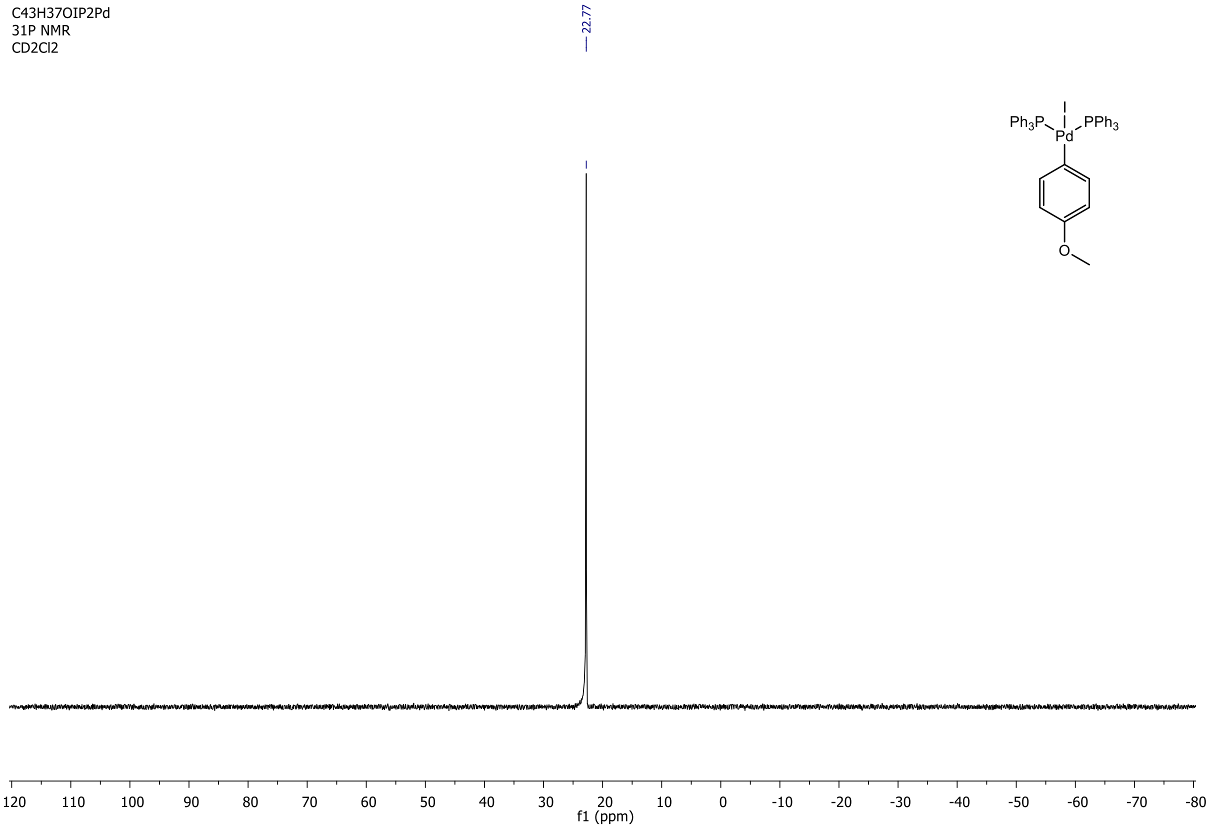

**Figure S3.** $^{31}$P NMR spectrum of **1**.

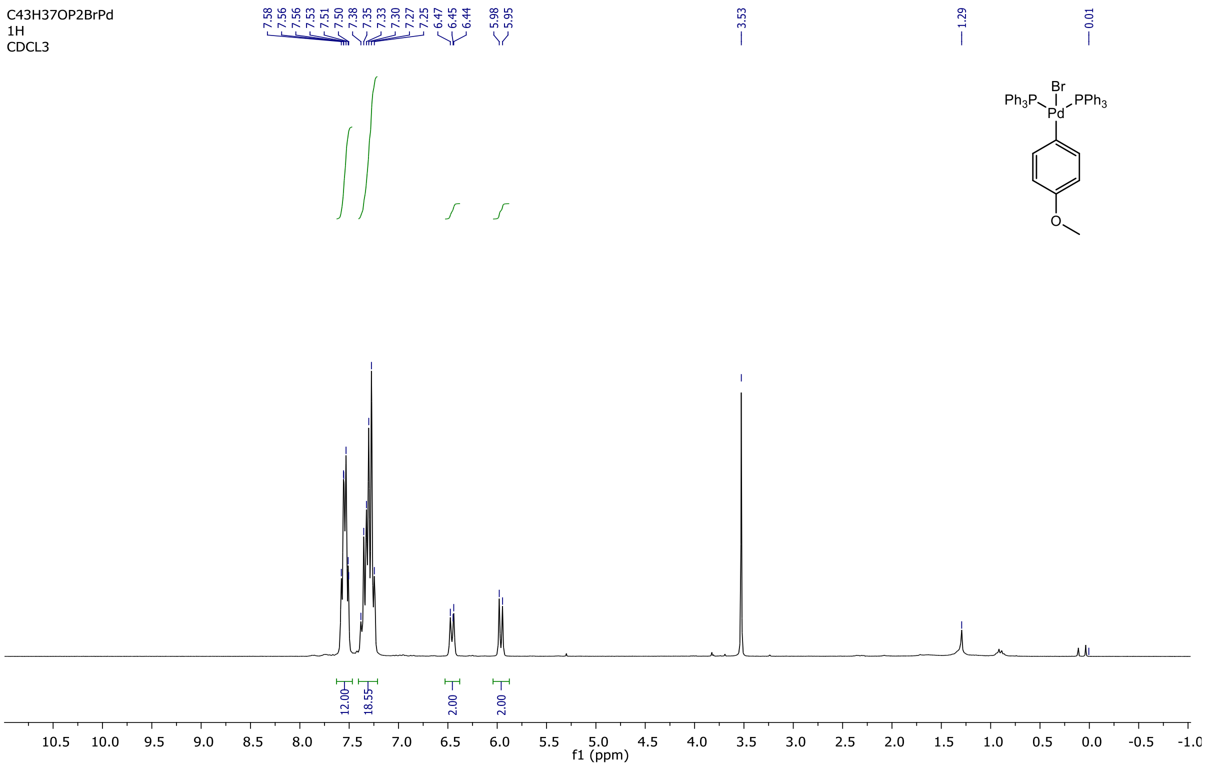


**Figure S4.** $^{1}$H NMR spectrum of **2.**

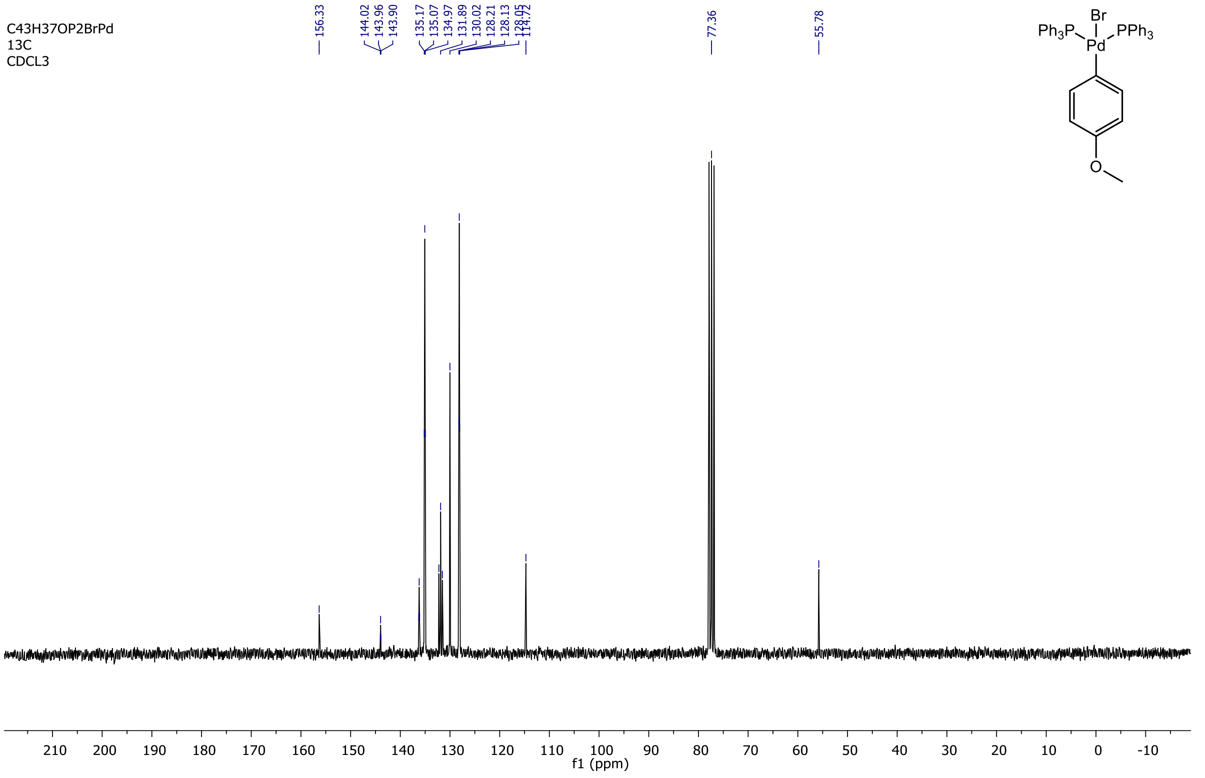


**Figure S5.** $^{13}$C NMR spectrum of **2.**

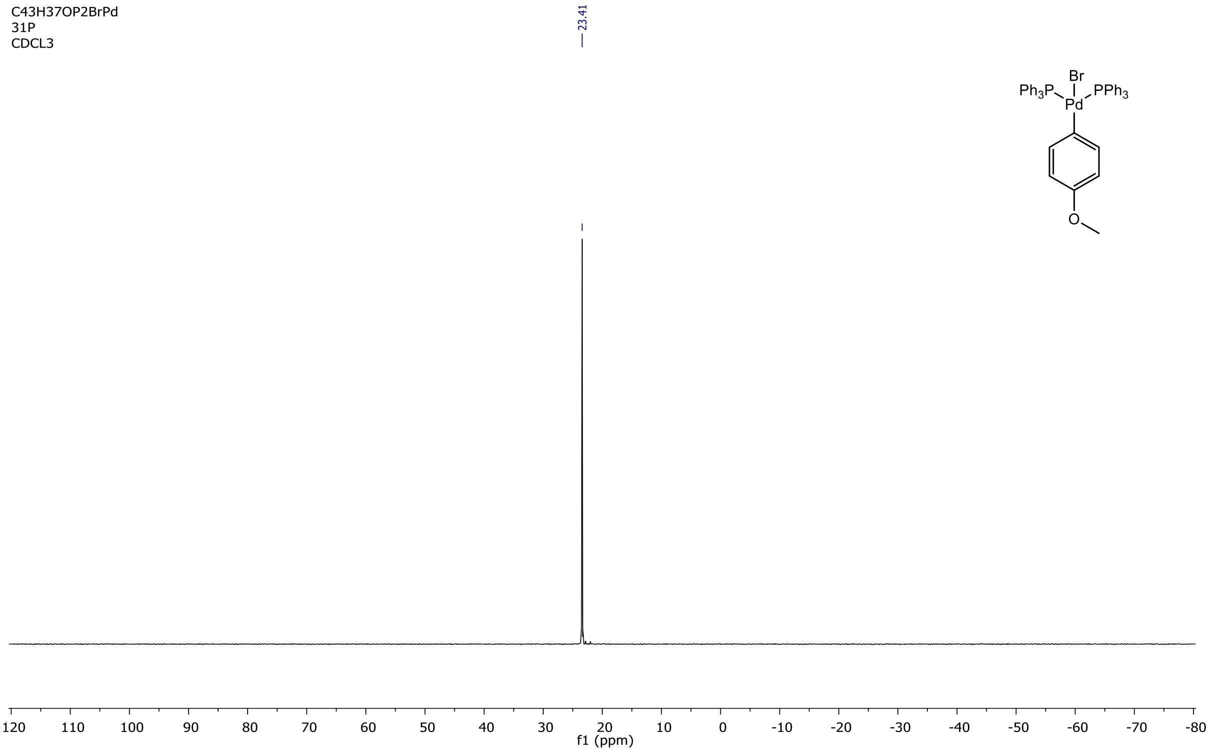


**Figure S6.** $^{31}$P NMR spectrum of **2**.

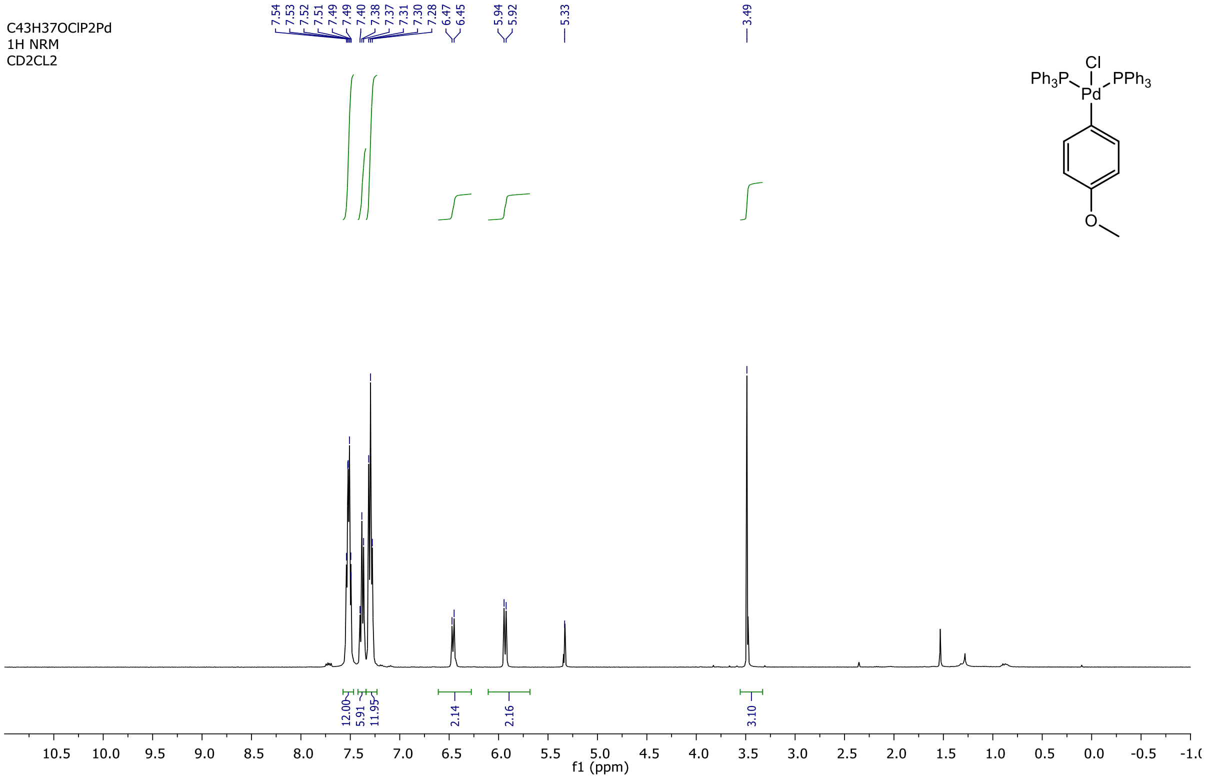

**Figure S7.** $^{1}$H NMR spectrum of **3**.

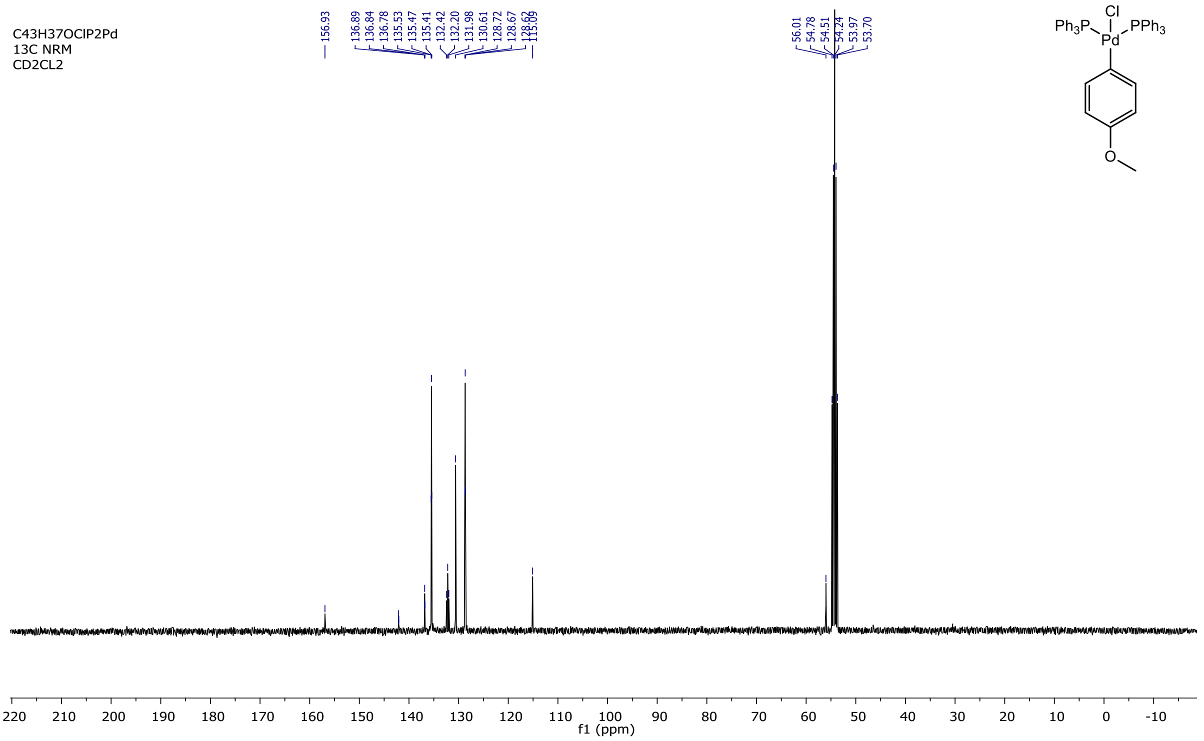


**Figure S8.** $^{13}$C NMR spectrum of **3.**

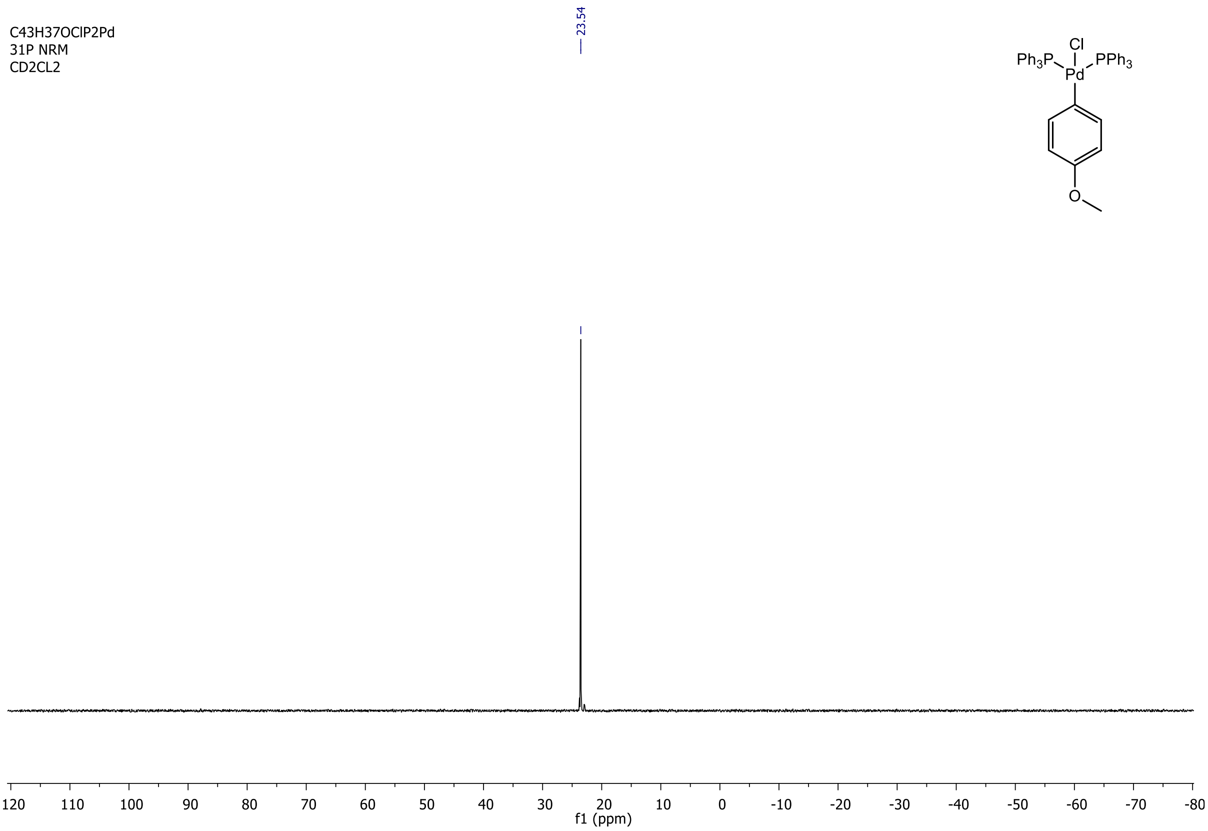


**Figure S9.** $^{31}$P NMR spectrum of **3**.

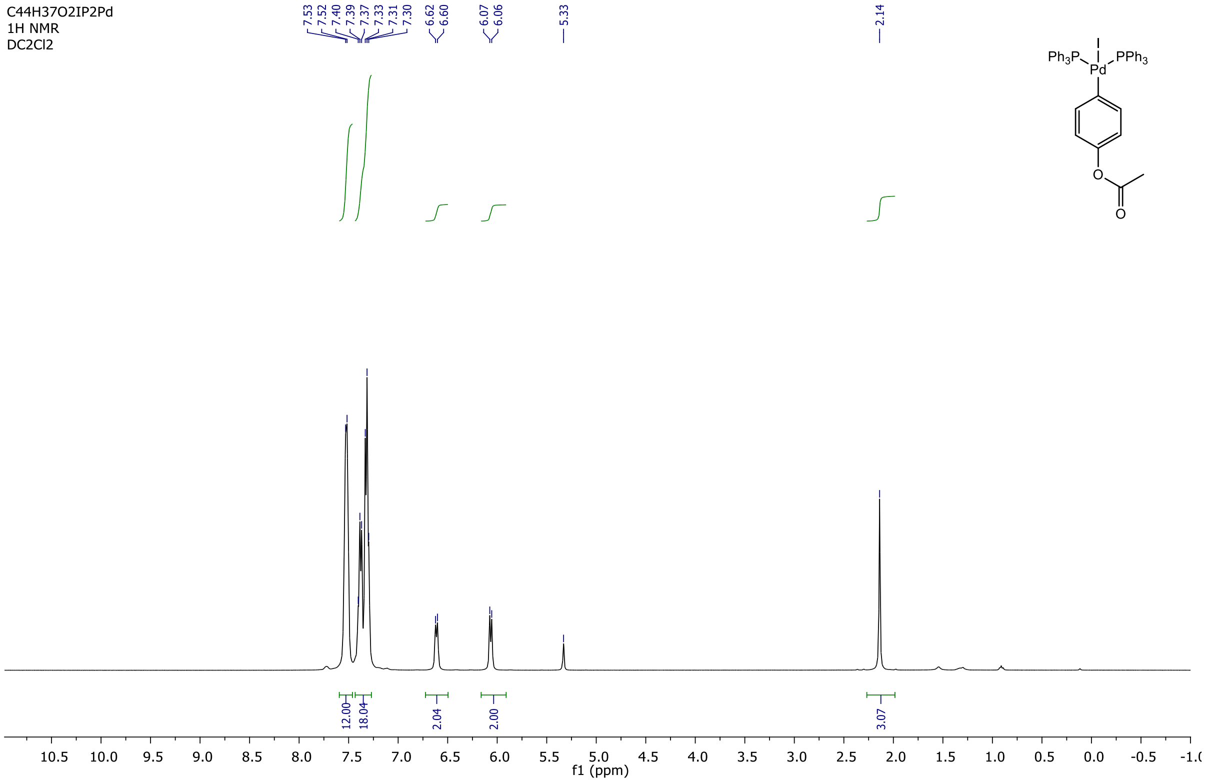


**Figure S10.** $^{1}$H NMR spectrum of **4**.

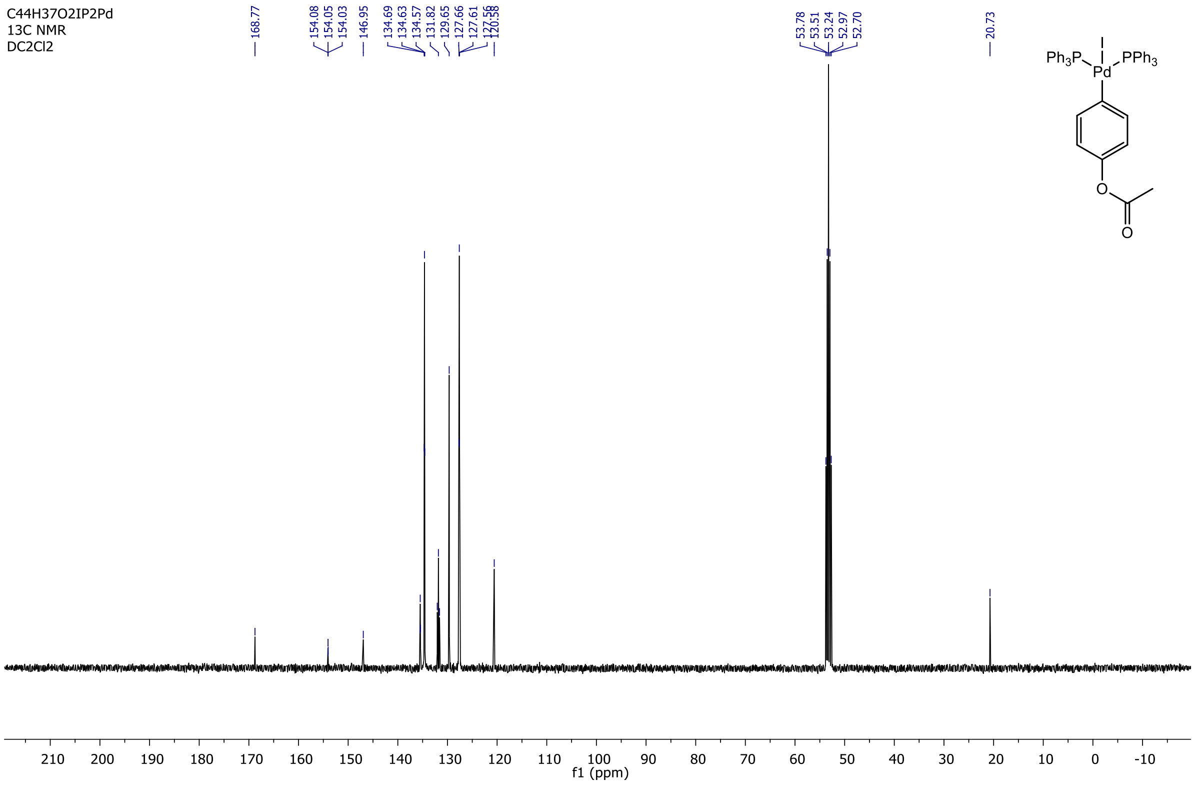


**Figure S11.** $^{13}$C NMR spectrum of **4.**

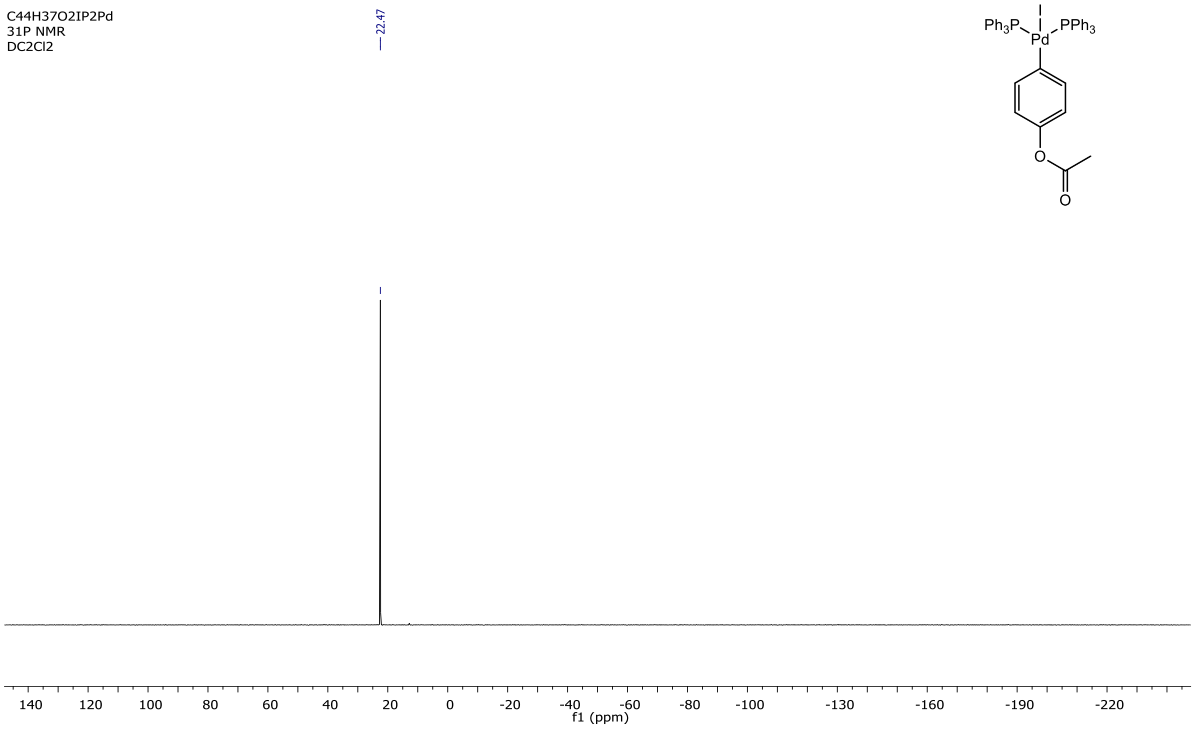


**Figure S12.** $^{31}$P NMR spectrum of **4**.

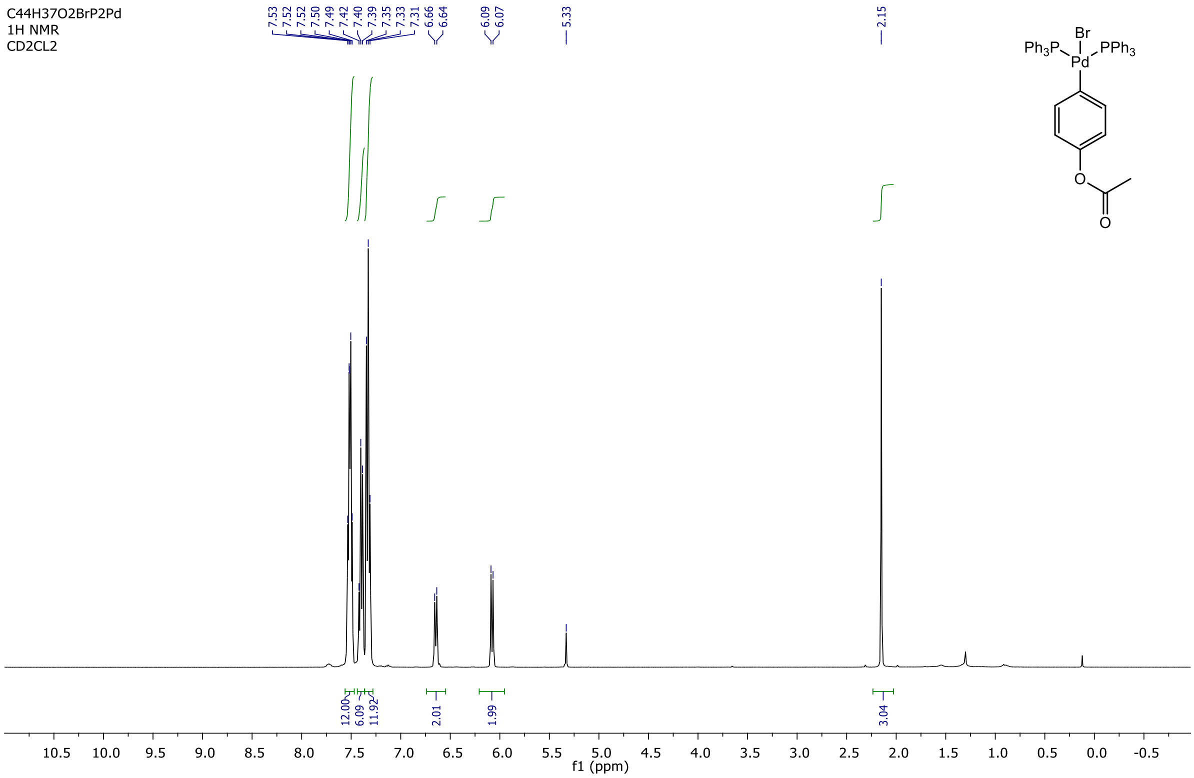


**Figure S13.** $^{1}$H NMR spectrum of **5**

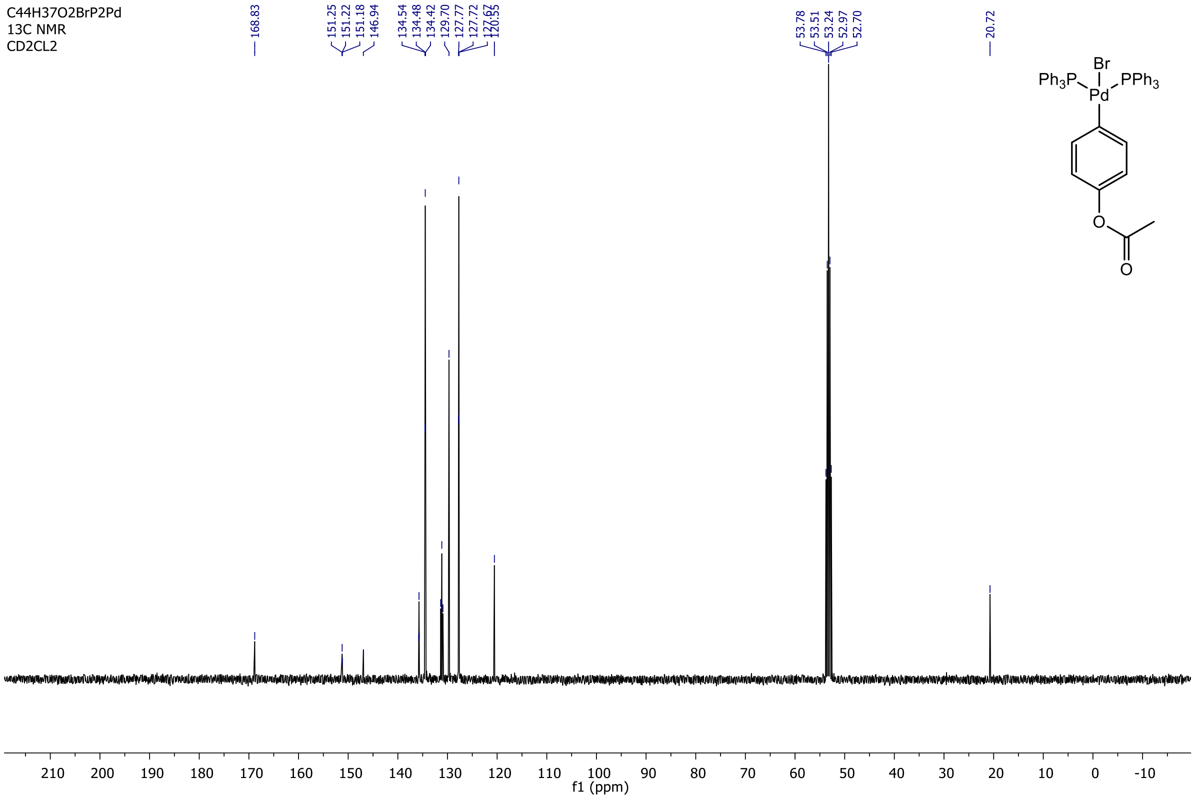


**Figure S14.** $^{13}$C NMR spectrum of **5**.

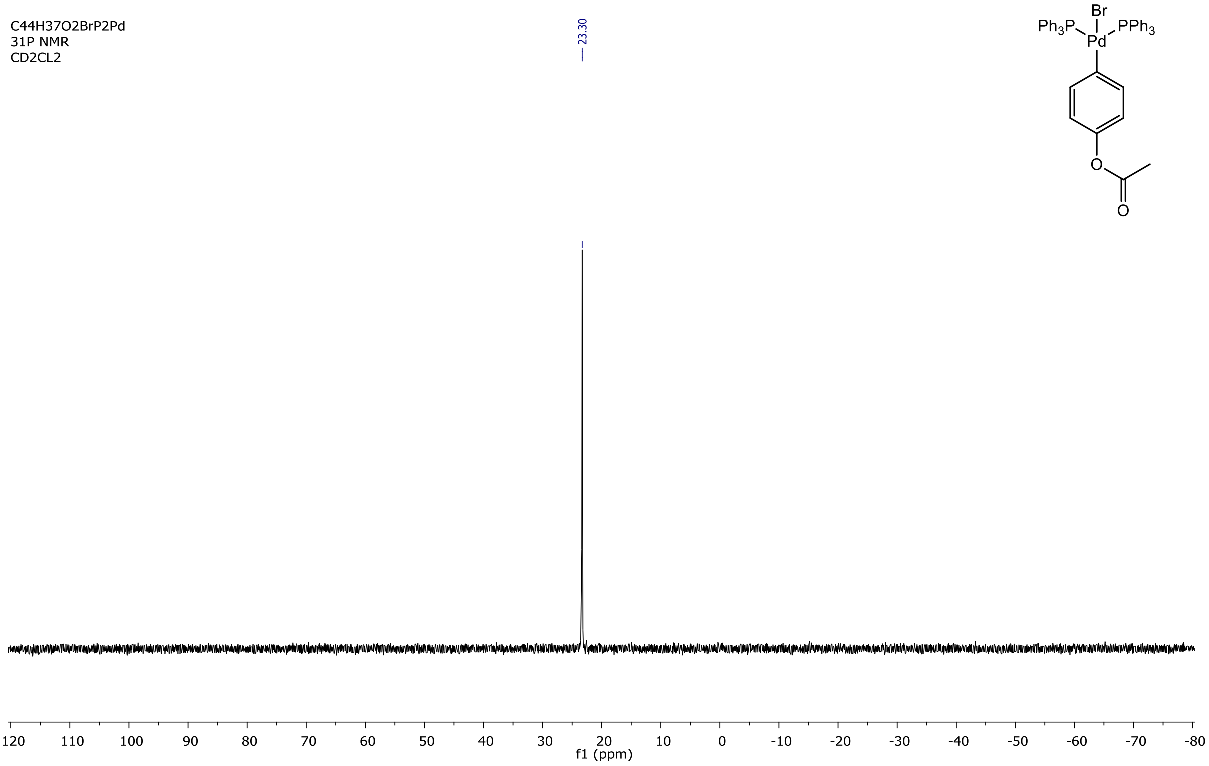


**Figure S15.** $^{31}$P NMR spectrum of **5**.

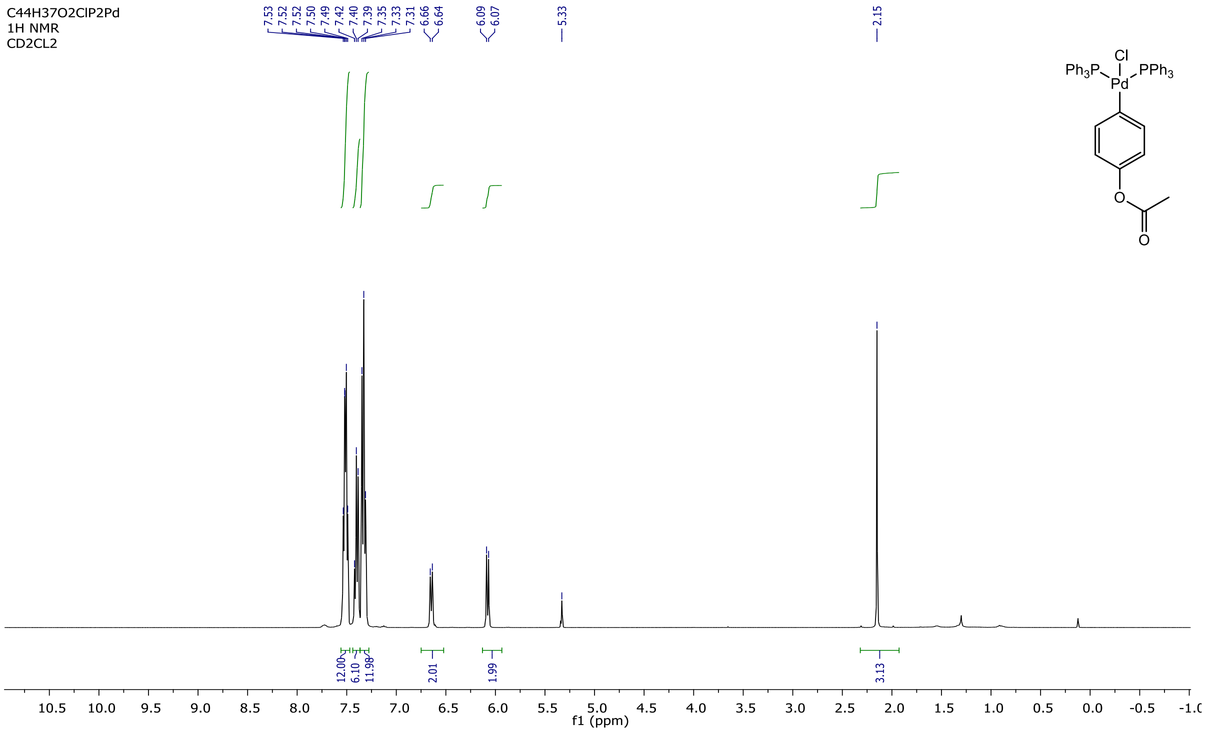


**Figure S16.** $^{1}$H NMR spectrum of **6**.

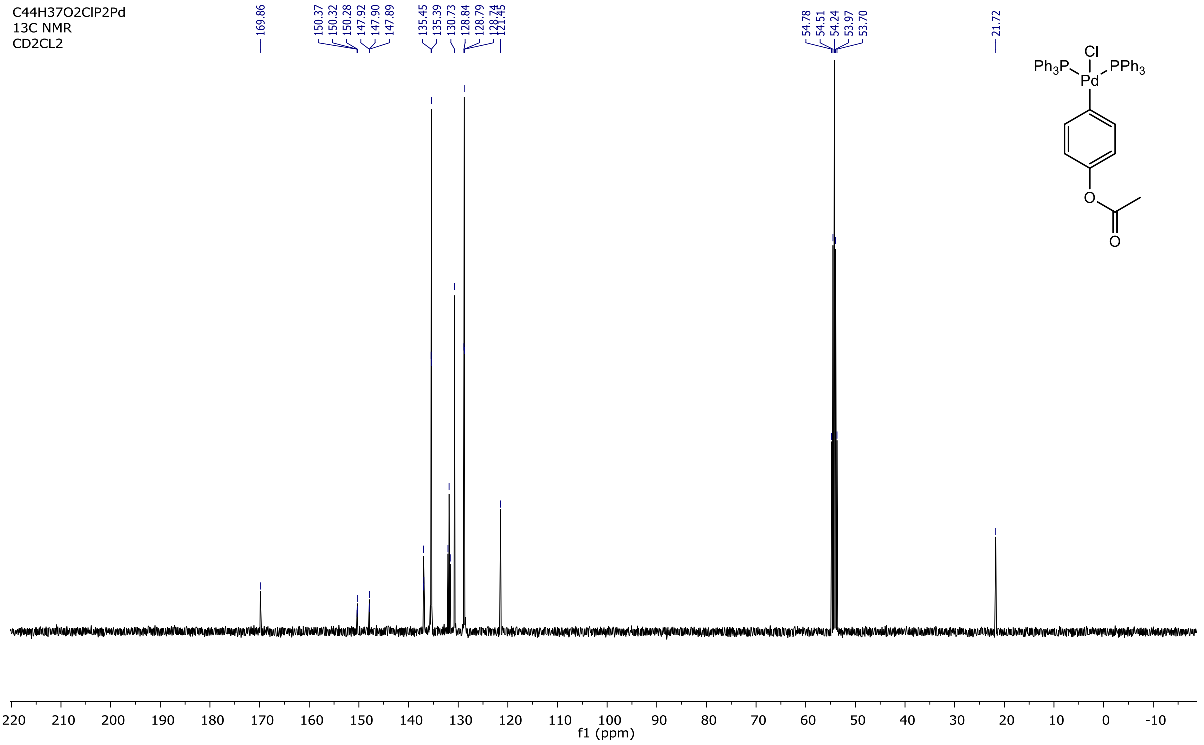


**Figure S17.** $^{13}$C NMR spectrum of **6.**

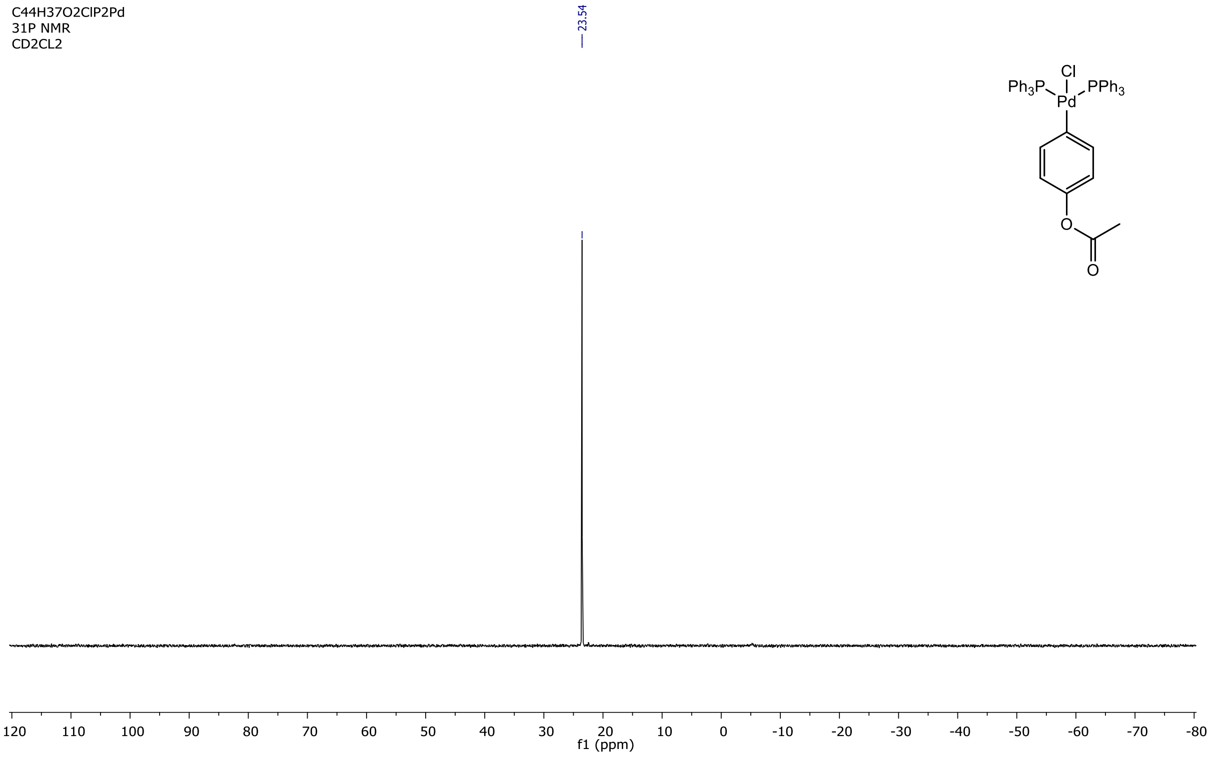


**Figure S18.** $^{31}$P NMR spectrum of **6**.

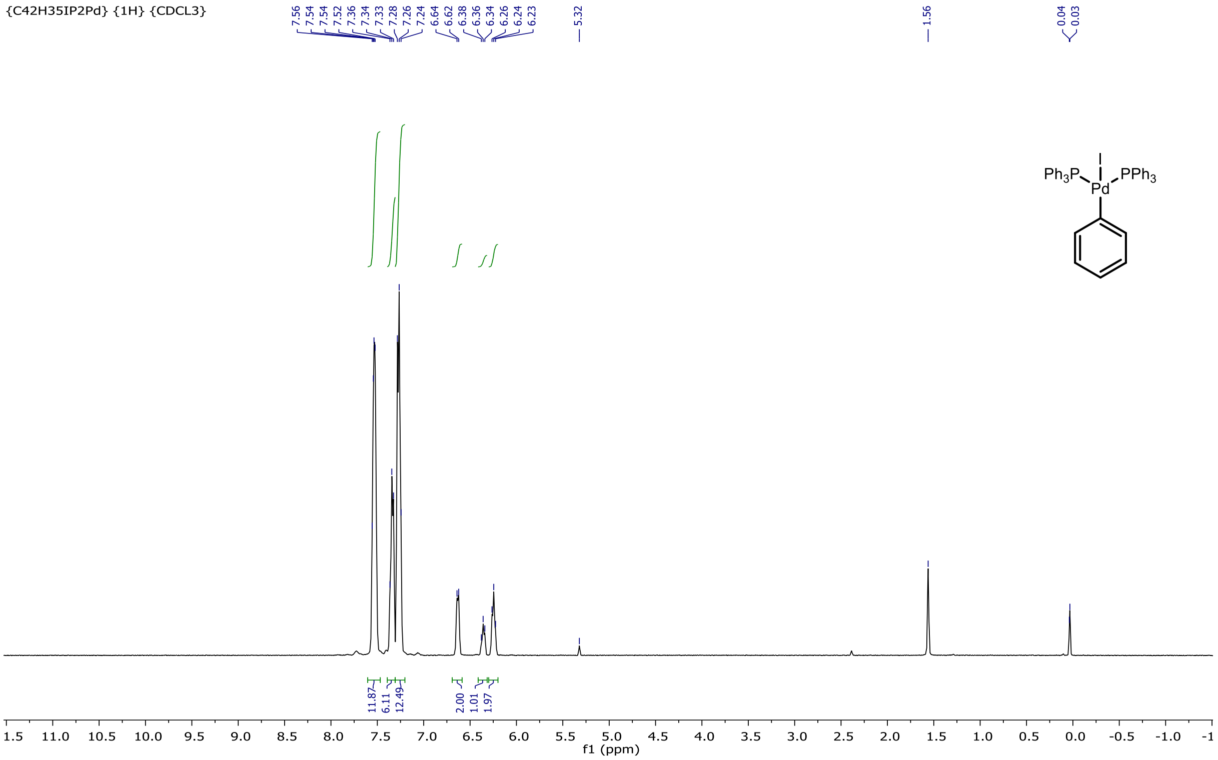


**Figure S19.** $^{1}$H NMR spectrum of **7.**

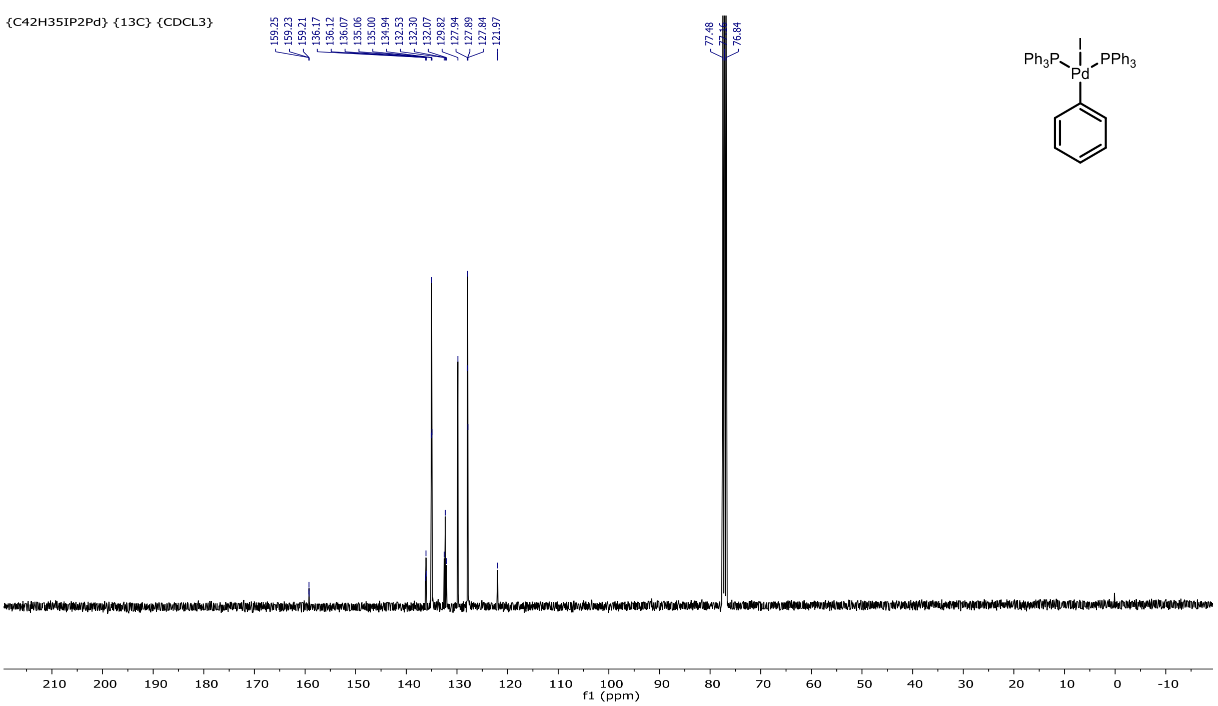


**Figure S20.** $^{13}$C NMR spectrum of **7.**

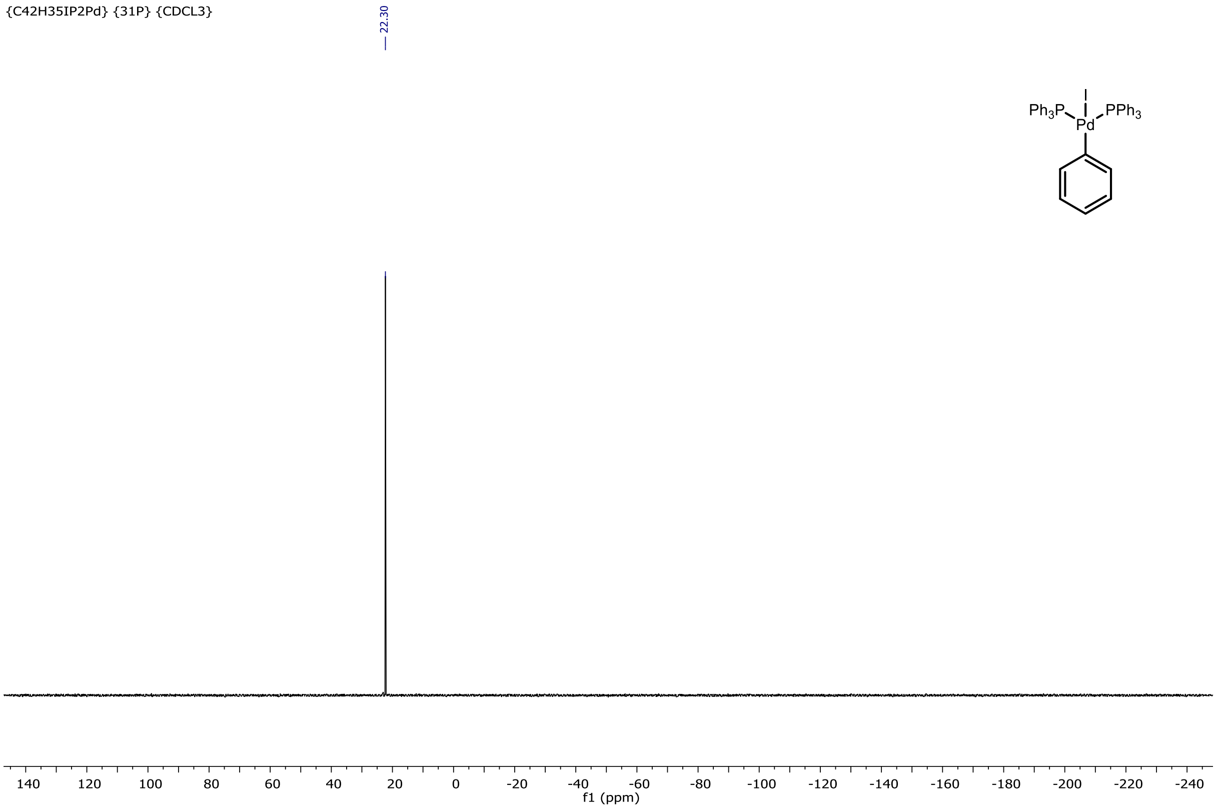


**Figure S21.** $^{31}$P NMR spectrum of **7**.

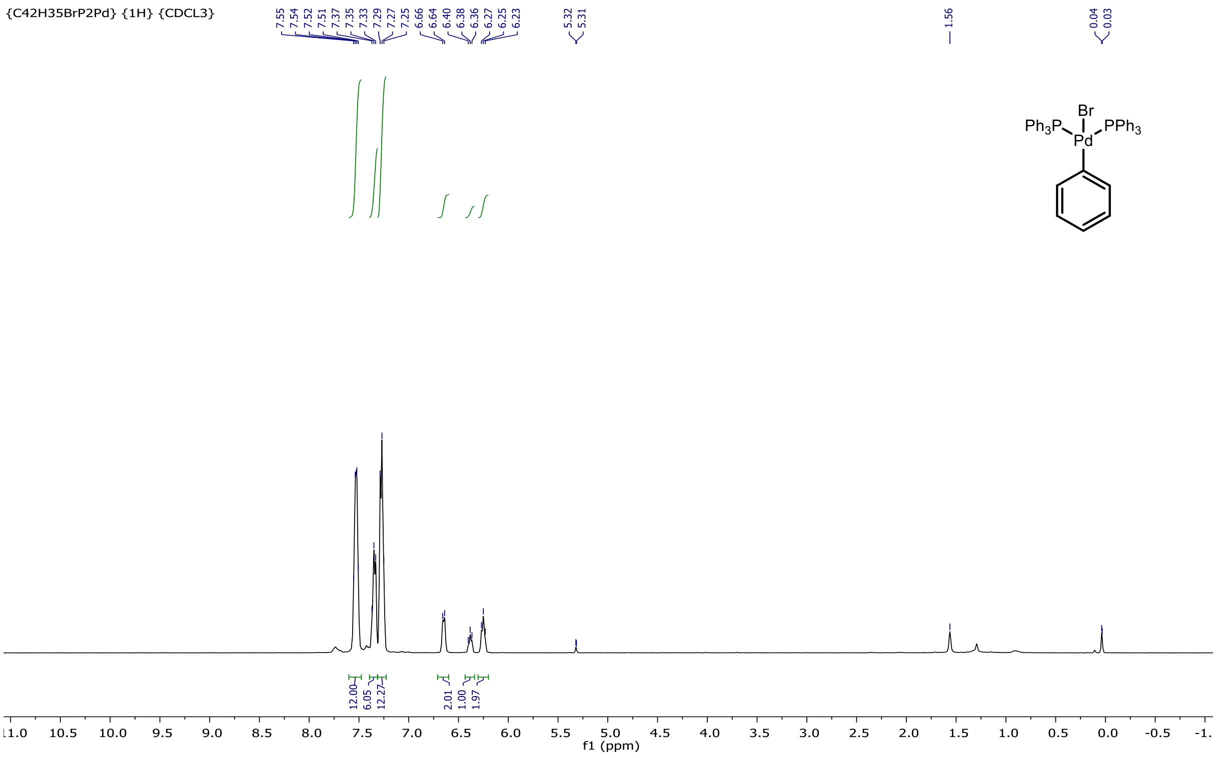


**Figure S22.** $^{1}$H NMR spectrum of **8.**

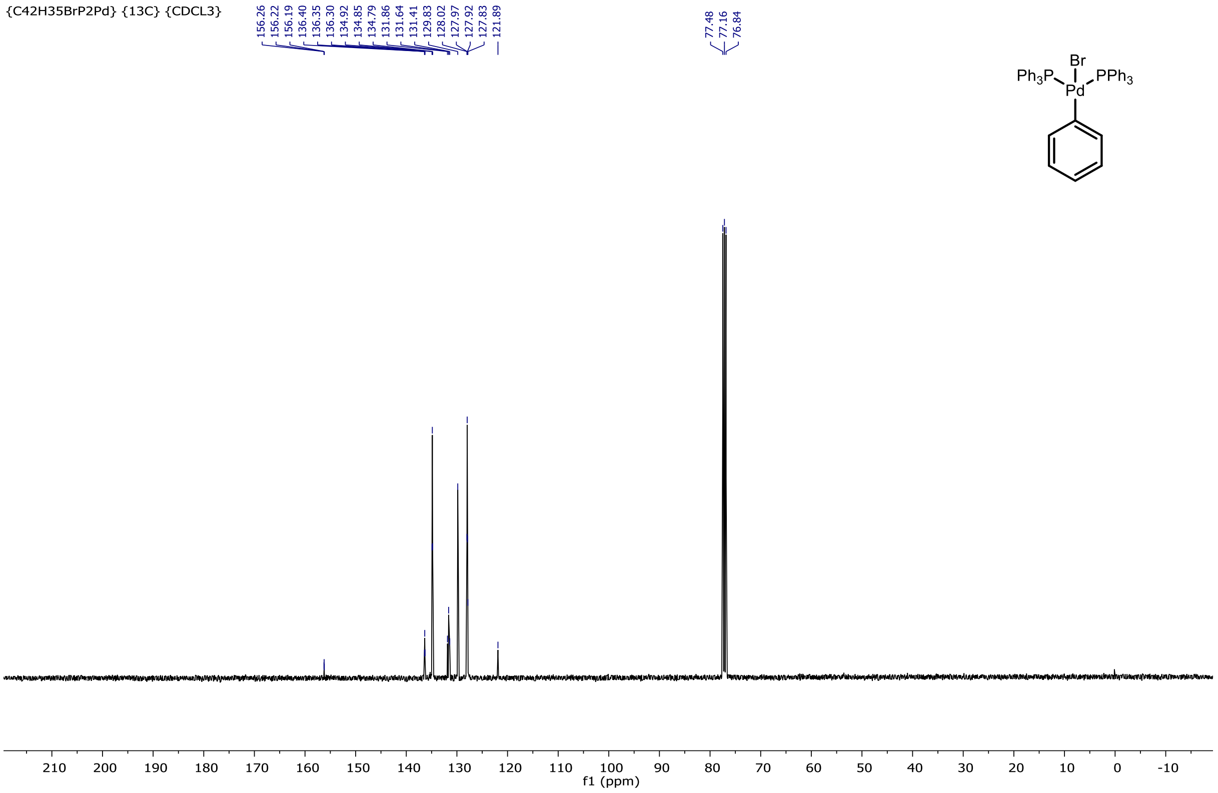


**Figure S23.** $^{13}$C NMR spectrum of **8.**

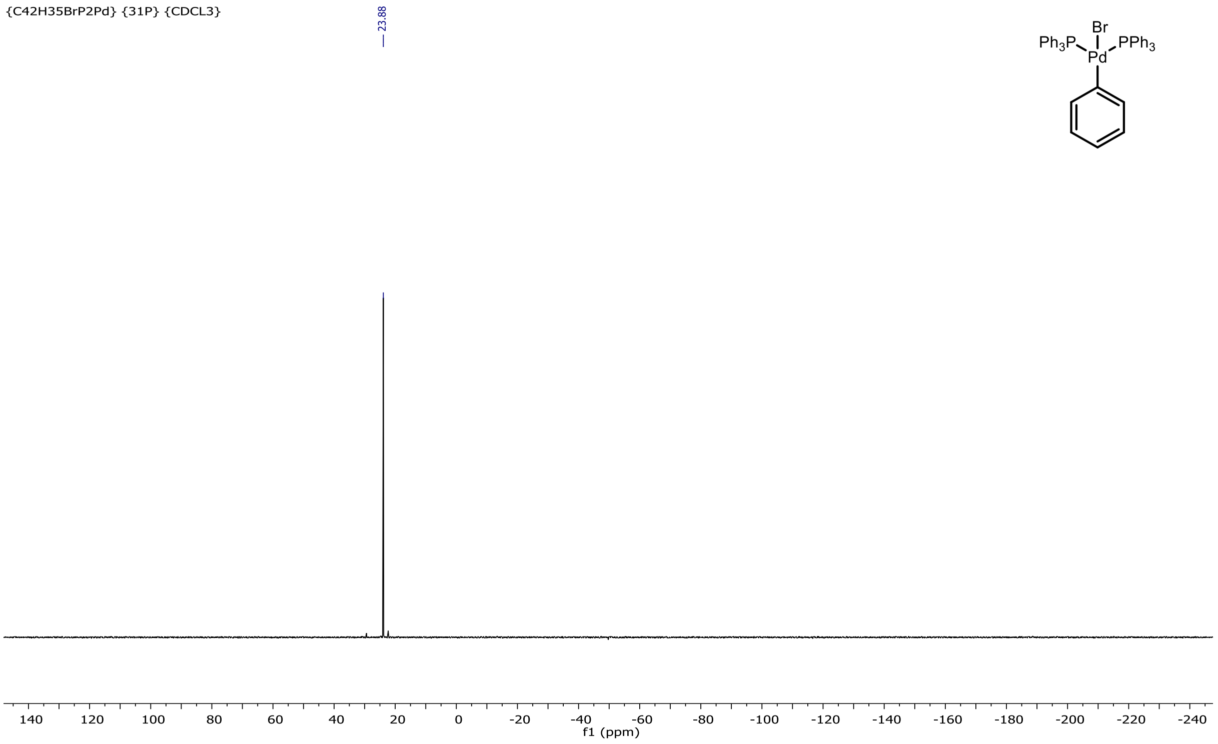


**Figure S24.** $^{31}$P NMR spectrum of **8**.

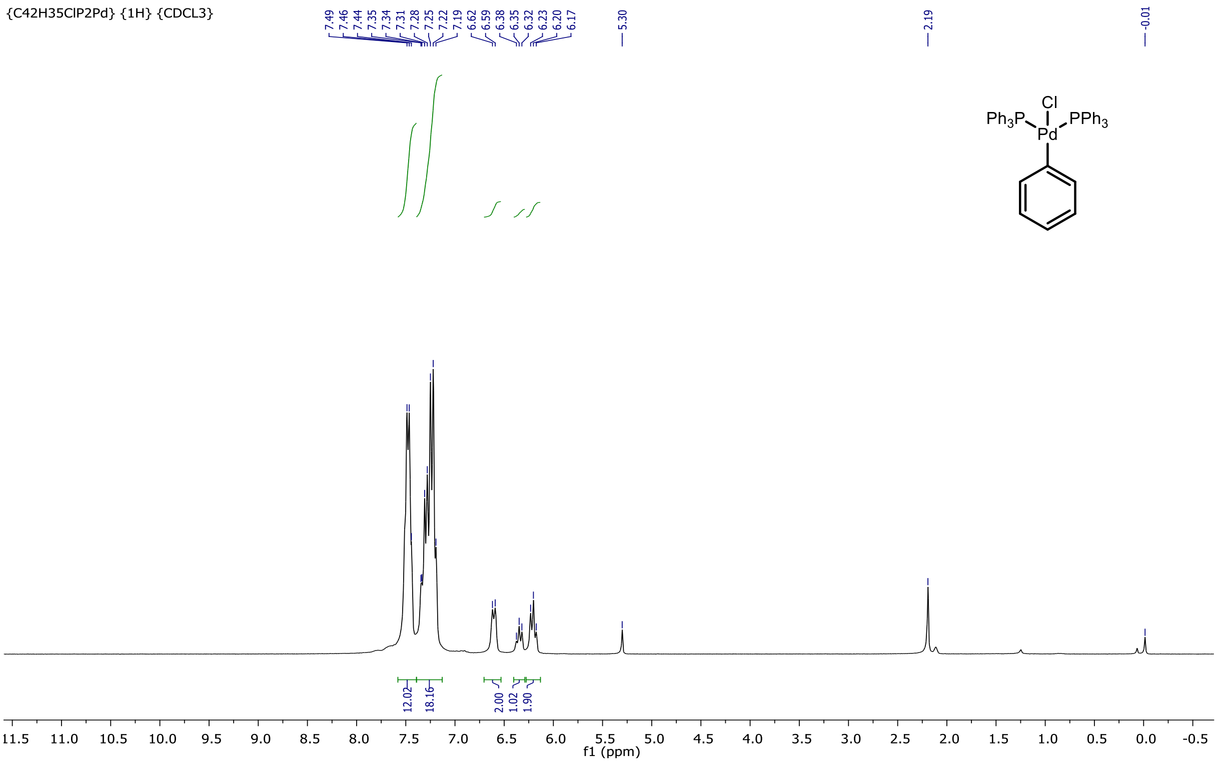


**Figure S25.** $^{1}$H NMR spectrum of **9**.

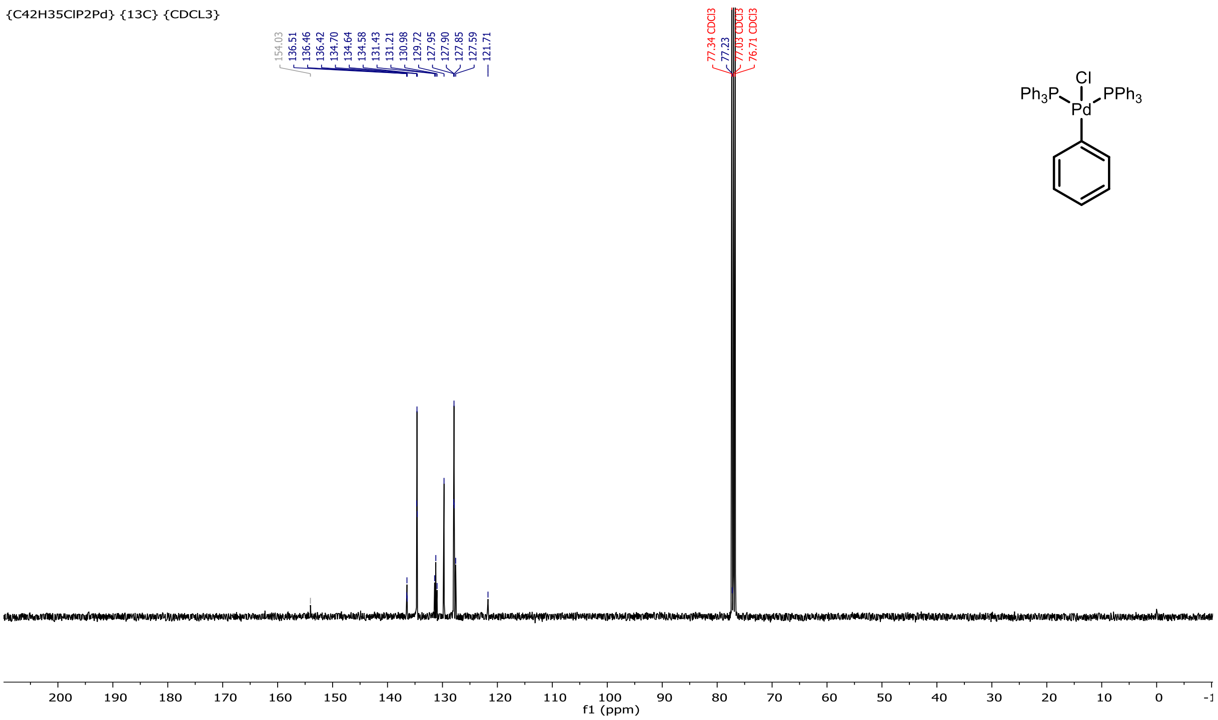


**Figure S26.** $^{13}$C NMR spectrum of **9.**

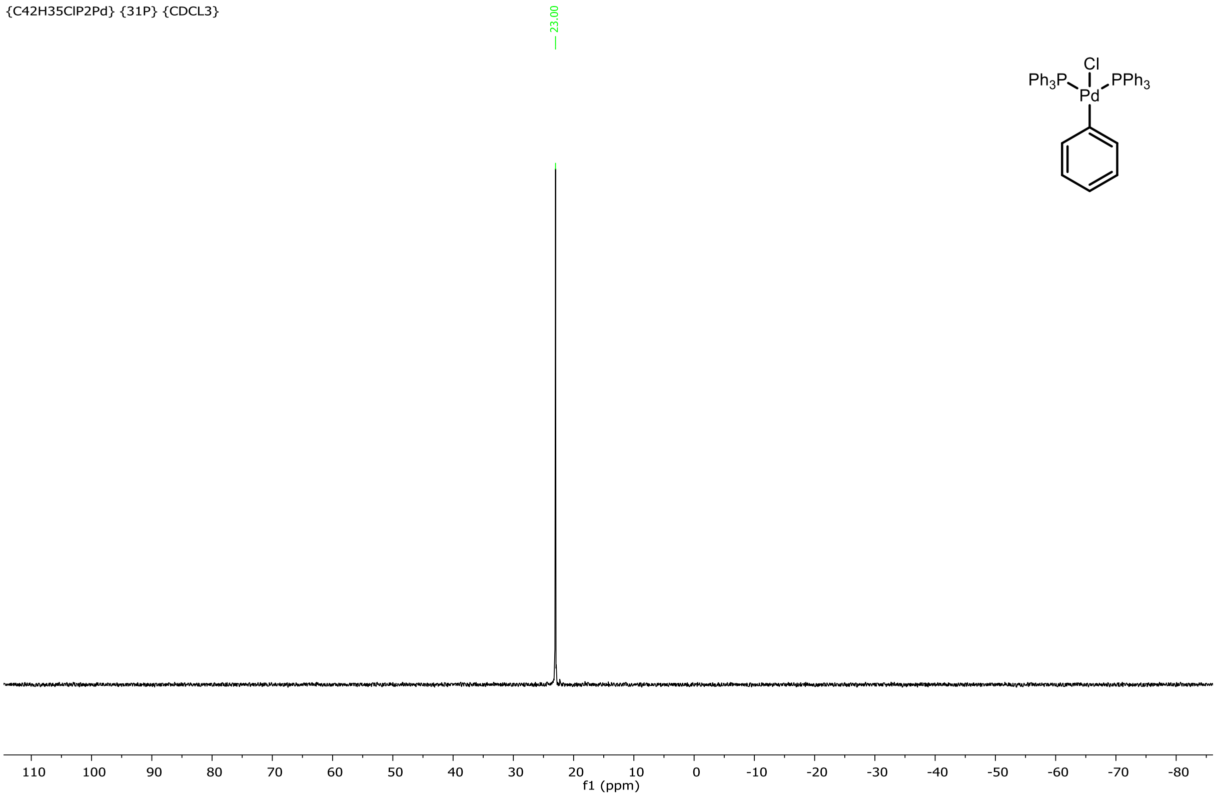


**Figure S27.** $^{31}$P NMR spectrum of **9**.

## 4. X-ray Crystal Structure Determination.

Crystal data were collected by applying the omega and phi scans method on a Bruker APPEX II or Smart CCD-1000 diffractometer using graphite-monochromated Mo-Kα radiation (λ = 0.71073 Å) from a fine-focus sealed tube source at 100 K. Computing data and reduction were made with the APPEX-II software [3]. The structure was solved using DIRDIF [4] and finally refined by full-matrix, least-squares based on $F^2$ by SHELXL [5]. An empirical absorption correction was applied using SADABS [6]. All non-hydrogen atoms were anisotropically refined and the hydrogen atom positions were included in the model by electronic density.
Crystallographic data have been deposited at the Cambridge Crystallographic Data Centre with the numbers CCDC-XXXXX (complex **1**), and CCDC-XXXXX (complex **2**). These data can be obtained, free of charge, from CCDC, 12 Union Road, Cambridge, CB2 1EZ, UK (fax: +44 1233 336033; e-mail: deposit@ccdc.cam.ac.uk; internet: http://www.ccdc.cam.ac.uk).

**Table S1**. Crystal data for compounds **1** and **2**

| Crystal data | **1** | **2** |
|---|---|---|
| Chemical formula | $C_{43}H_{37}OIP_2Pd$ | $C_{43}H_{37}OBrP_2Pd$ |
| Mr | 864.96 | 817.97 |
| Crystal system, space group | Monoclinic, *Ia* | Orthorhombic, *Pbca* |
| Temperature (K) | 100 | 100 |
| *a* (Å) | 11.3164 (7) | 11.4818 (11) |
| *b*(Å) | 13.5866 (8) | 23.717 (3) |
| *c* (Å) | 23.3413 (16) | 26.133 (3) |
| $\beta$(°) | 94.306 (3) | - |
| $V$ (Å$^3$) | 3578.6 (4) | 7116.3 (13) |
| *Z* | 4 | 8 |
| Radiation type | Mo Kα radiation | Mo Kα radiation |
| $\mu$ (mm$^{-1}$) | 1.50 | 1.77 |
| Crystal size (mm) | 0.22 × 0.20 × 0.11 | 0.27 × 0.08 × 0.06 |
| Data collection | | |
| Diffractometer | BRUKER APPEX-II CCD | BRUKER APPEX-II |
| Absorption correction | Multi-scan<br>BRUKER SADABS2012/1 | Multi-scan<br>SADABS2016/2 - Bruker AXS area detector scaling and absorption correction |
| *Tmin, Tmax* | 0.765, 0.825 | 0.693, 0.801 |
| No. of measured, independent and observed *[I > 2σ(I)]* reflections | 64342, 8854, 8583 | 101525, 6743, 5008 |
| $R_{\mathrm{int}}$ | 0.056 | 0.117 |
| (sin θ/λ)max (Å$^{-1}$) | 0.667 | 0.610 |
| Refinement | | |
| R[F2 > 2σ(F2)], wR(F2), S | 0.022, 0.048, 1.04 | 0.041, 0.107, 1.06 |
| No. of reflections | 8854 | 6743 |
| No. of parameters | 435 | 434 |
| No. of restraints | 2 | - |
| H-atom treatment | H-atom parameters constrained | H-atom parameters constrained |

| | | |
|---|---|---|
| $\Delta\rho_{max}$, $\Delta\rho_{min}$ (e Å$^{-3}$) | 0.56, −0.30 | 0.64, −1.38 |

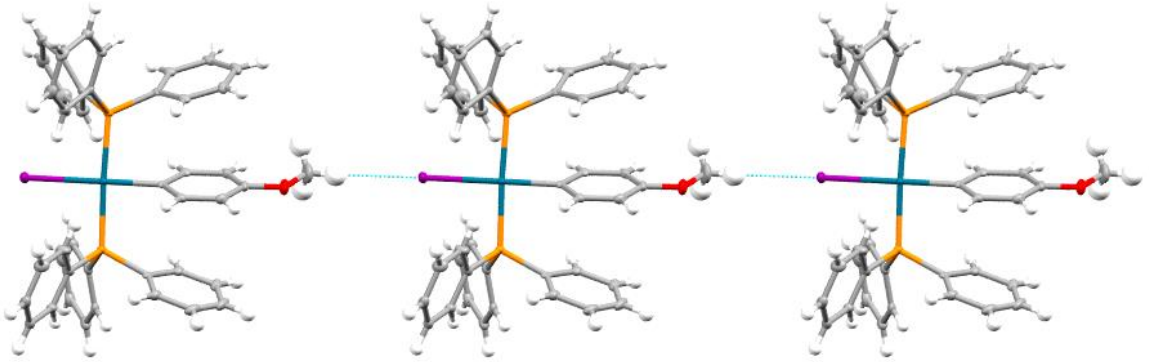

**Figure S28.** ORTEP representation of **1** with thermal ellipsoids drawn at the 50% probability level showing I—H interactions.

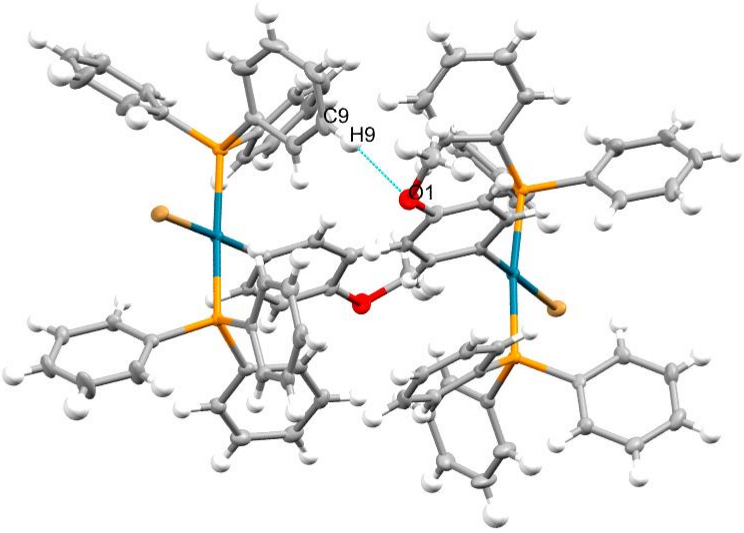


**Figure S29.** ORTEP representation of **2** with thermal ellipsoids drawn at the 50% probability level showing O—H interactions.

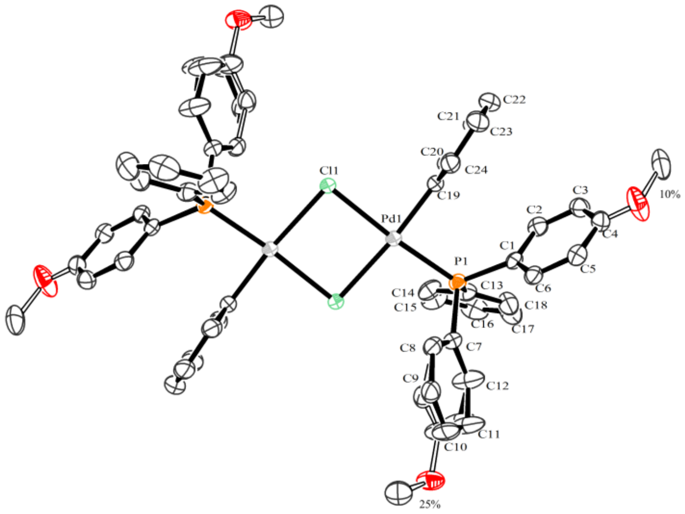


**Figure S30**. ORTEP representation of byproduct isolated from complex **3** solution resulting from self-decomposition/rearrangement reaction.

## 5. References

[1] K.S. Etsè, F. Boschini, C. Karegeya, E. Roex, G. Zaragoza, A. Demonceau, R. Cloots, A. Mahmoud, Electrochim. Acta 337 (2020), 135659.

[2] G.R. Fulmer, A.J.M. Miller, N.H. Sherden, H.E. Gottlieb, A. Nudelman, B.M. Stoltz, J.E. Bercaw, K.I. Goldberg, NMR chemical shifts of trace impurities: Common laboratory solvents, organics, and gases in deuterated solvents relevant to the organometallic chemist, Organometallics. 29 (2010) 2176–2179. doi:10.1021/om100106e.

[3] Bruker, APPEX-II, Bruker AXS Inc., Madison, WI, USA, 2004.

[4] P.T. Beurskens, G. Admiraal, G. Beurskens, W.P. Bosman, S. Garcia-Granda, R.O. Gould, J.M.M. Smits, C. Smykalla, DIRDIF92: The DIRDIF Program System, Technical Report of the Crystallography Laboratory, 1992.

[5] G.M. Sheldrick, SHELX97 (SHELXS97 and SHELXL97), Programs for Crystal Structure Analysis, University of Göttingen, Göttingen, Germany, 1997.

[6] G.M. Sheldrick, SADABS, Programs for Scaling and Correction of Area Detection Data, University of Göttingen, Göttingen, Germany, 1996.
